# Clinical manifestations, prognostic impact, and relapse in polyarteritis nodosa: a systematic review and meta-analysis

**DOI:** 10.1007/s00296-026-06082-8

**Published:** 2026-02-19

**Authors:** Nikolaos Taprantzis, Maria Eleni Kasimeri, Dimosthenis Chrysikos, Amir Shihada, Alexandros Samolis, George Tsakotos, Martina Liga, Theodore Troupis

**Affiliations:** 1 Department of Anatomy, Athens Medical School, National and Kapodistrian University of Athens, Mikras Asias 75, 11527 Athens, Greece; 2 School of Medicine, Medicine and Surgery, University of Palermo, Palermo, Italy

**Keywords:** Polyarteritis nodosa, Prognosis, Signs and symptoms, Vasculitis, Mortality

## Abstract

Polyarteritis Nodosa is a rare necrotizing vasculitis with a broad and variable clinical presentation, driven by its ability to affect multiple organs and systems. This heterogeneity influences disease severity, relapse risk, and mortality, highlighting the prognostic importance of its diverse manifestations. This systematic review and meta-analysis synthesize the available evidence to define the clinical profile of Polyarteritis Nodosa and highlight key prognostic factors. A systematic search of electronic databases was conducted to identify studies reporting clinical manifestations and outcomes of patients diagnosed with Polyarteritis Nodosa. Pooled prevalence summary estimates were calculated using R software, including subgroup analyses by age group. Case Fatality Rate was calculated to determine the clinical severity of the manifestations. Across adult and pediatric groups, the most common manifestations were fever (~ 52%), myalgia (~ 53%), and cutaneous involvement (~ 56%). Age significantly moderated the prevalence of fever, arthralgia, hypertension, and peripheral neuropathy. Several symptoms were frequently observed among patients who experienced relapse, particularly cutaneous involvement (~ 66%) and myalgia (~ 64%). While cardiac involvement was associated with higher case fatality, gastrointestinal manifestations accounted for a greater proportion of reported deaths overall. Total mortality was approximately 13%, relapse occurred in about 27% of patients, while roughly 65% of patients achieved remission. This systematic review with meta-analysis provides crucial information to clinical physicians, regarding the clinical profile and the prognostic factors of Polyarteritis Nodosa. Thus, these insights could guide management strategies and increase the survival and remission rate of these patients. PROSPERO: https://www.crd.york.ac.uk/PROSPERO/view/CRD420251249274.

**Supplementary Information:**

The online version contains supplementary material available at 10.1007/s00296-026-06082-8.

## Introduction

Polyarteritis nodosa (PAN) is recognized as a form of necrotizing vasculitis that predominantly affects medium-sized arteries, although smaller vessels may also be involved, albeit less frequently [1]. The clinical spectrum of PAN is notably broad, reflecting its potential to affect multiple organ systems. Patients may present with a combination of organ-specific manifestations as well as a collection of nonspecific constitutional symptoms, including fever, malaise, and weight loss [1, 2]. According to the existing literature and previous studies, over 90% of all affected patients will develop some form of systemic manifestation, such as fever, weakness or weight loss. Neurologic involvement most commonly presents as peripheral neuropathy, and more often in the form of mononeuritis multiplex. Cutaneous involvement, observed in around 60% of cases, typically manifests as subcutaneous nodules, palpable purpura, or, in more severe cases, necrotic ulcers [1, 3,4]. It should be noted that a distinct subset of polyarteritis nodosa, namely cutaneous PAN, is characterized by disease manifestations confined to the skin [1]. Less common organ system involvement, such as renal involvement, typically results from stenosis or aneurysm formation of the renal vessels and manifests clinically as hypertension, hematuria, and/or proteinuria [3–6]. As far as gastrointestinal manifestations are concerned, the clinical spectrum is more diverse, with patients presenting more often with abdominal pain, ischemia, perforation, hemorrhage, and even pancreatitis. Finally, for cardiovascular involvement, affected individuals are reported to experience myocardial infarction and/or heart failure [1, 3, 4].

Despite its clinical significance, PAN remains a rare disease, with prevalence in European populations reported to range between 2 and 31 cases per million individuals. Epidemiological data further suggest that middle-aged men are more frequently affected compared with other demographic groups [7]. Pediatric cases, on the other hand, are relatively uncommon, and available data indicate that the disease is likely less prevalent among younger populations [8].

This systematic review with meta-analysis aims to provide prevalence data regarding the main manifestations of polyarteritis nodosa, as well as investigate the severity of each one by assessing its contribution to relapse and mortality.

##  Materials and methods

### Study selection

We systematically searched through the PubMed, Embase, Web of Science, and Scopus databases using the following MeSH terms: “Polyarteritis Nodosa” AND (“prognosis” OR “Risk Factors” OR “recurrence” OR “signs and symptoms” OR “mortality” OR “Disease Attributes” OR “prevalence”, order to collect data that would fit the criteria of our review. This database search took place from 5th of June 2025 to 12th of September 2025. Studies published in English, with no date restrictions were eligible for screening. Since this paper used only published data, any Institutional review Board approval or any other consent were not needed.

Three reviewers (DC, NT and ML) independently screened the titles and abstracts of all records retrieved through the database searches. Studies that appeared relevant were then reviewed in full text. Disagreements at either stage were resolved through discussion and consensus between the two reviewers. No automation tools or machine learning algorithms were used in the selection process. Duplicates were removed manually using Microsoft Excel. This systematic review with meta-analysis adhered to Gasparyan et al. recommendation on comprehensive search strategies [9].

### Inclusion criteria

The inclusion of studies, that were analyzed in this review, was based on a fixed collection of inclusion and exclusion criteria. Studies were included if (1) participants had been diagnosed with Polyarteritis Nodosa, (2) manifestations/symptoms of the patients were reported, (3) the disease outcomes were noted down.

During our literature search, all types of polyarteritis nodosa were included, meaning that systemic, cutaneous, pediatric, adult, monogenic, and HBV-associated PAN, were all included.

Regarding disease classification, we accepted established diagnostic frameworks commonly used for the classification of Polyarteritis Nodosa. Specifically, studies were eligible if they applied recognized criteria such as the 1990 American College of Rheumatology (ACR) classification criteria, the Chapel Hill Consensus Conference (CHCC) definitions (1994 or 2012), the French Vasculitis Study Group (FVSG) criteria, or the European League Against Rheumatism (EULAR)/Paediatric Rheumatology European Society (PRES)/Paediatric Rheumatology International Trials Organization (PRINTO) recommendations [10–13].

In studies that did not explicitly apply an established classification system, inclusion was still permitted when the diagnosis of PAN was clearly defined by the study authors. Specifically, these studies were accepted if PAN was diagnosed based on compatible clinical manifestations together with supportive laboratory findings and/or histopathological evidence of necrotizing vasculitis.

These inclusion criteria were established to provide a consistent and reliable reference framework, minimizing confusion and reporting discrepancies across studies.

### Exclusion criteria

Studies were excluded if (1) participants experienced more than one disease at the same time, (2) no disease outcomes were reported, (3) animal studies or conference abstracts.

### Data extraction

Three reviewers (DC, NT, and ML) independently extracted data from the included studies using a standardized data extraction form in Microsoft Excel. The extracted data included raw count data for Polyarteritis Nodosa manifestations, disease outcomes, manifestation-related deaths, and disease outcomes. After completing data extraction independently, the reviewers compared their datasets and resolved discrepancies through consensus. No automated tools were used in the data extraction process.

### Data sought

The primary outcomes of interest were the clinical manifestations of Polyarteritis Nodosa, such as fever, weight loss, arthralgia, myalgia, hypertension, cutaneous involvement, hypertension, gastrointestinal involvement, cardiac involvement, asthma, central nervous system involvement, peripheral neuropathy, renal involvement, mean disease duration (in months), and mean follow-up duration (in months). Secondary outcomes included disease reported outcomes, like mortality, remission and relapse. Finally, the number of deaths and relapses that each manifestation caused or contributed to, was also obtained.

Regarding the definitions of the manifestations, studies reported symptoms according to the definitions established in the classification systems they used for the disease diagnosis. In our review, we accepted and harmonized manifestations based on these standardized definitions from widely recognized frameworks, including the 1990 ACR criteria, the 1994/2012 CHCC definitions, the French Vasculitis Study Group (FVSG) criteria, and EULAR/PRES/PRINTO recommendations [10–13].

When studies did not explicitly reference a formal classification system, we included their reported clinical manifestations as long as the diagnosis of PAN was supported by accepted diagnostic elements, namely compatible clinical features together with at least one objective indicator such as histopathological confirmation of necrotizing vasculitis, characteristic angiographic findings, or other supportive laboratory/imaging evidence.

After an initial search of the literature, regarding the most common clinical manifestations of Polyarteritis Nodosa, it was decided that this group of patient symptoms, that represented different categories of disease features (such as neurological, cardiovascular, gastrointestinal) would be included [1, 7]. The goal was to explore the gravity and clinical associations between disease and patient characteristics and the eventual outcomes of the disease. This investigation aimed to provide information regarding the general picture of risk and prognostic factors of Polyarteritis Nodosa. To minimize variability across studies, we applied unified working definitions for relapse and remission during data extraction. Relapse was defined as the recurrence or re-appearance of one or more PAN-related clinical manifestations after a documented period of remission. Remission was defined as the attenuation or disappearance of clinical manifestations attributable to PAN, as reported by the original study authors. For each included study, we aimed to extract all available results related to the predefined outcome domains. If multiple time points or subgroups were reported, we prioritized baseline data. Where outcomes were reported using different terms or formats, data were standardized based on consistent definitions across studies.

### Other variables extracted

In addition to the primary and secondary outcomes, we extracted key study characteristics, including first author, year of publication, and total number of participants. Furthermore, patient age group was extracted so as to investigate any differences in pooled prevalence between the different patient groups. Two groups were created, one for adults and one for pediatric patients.

### Risk of bias assessment

Risk of bias for each included study was assessed using the Quality in Prognostic Studies (QUIPS) Tool [14]. Two reviewers (DC and NT) conducted the assessments independently. Each domain of the tool was rated separately, and an overall judgment of risk (low, moderate, or high) was assigned per study. In cases of disagreement, a consensus was reached through discussion.

### Statistical methods

We performed statistical analyses using R (version 4.3.2) and RStudio, utilizing the “meta”, “metafor”, and “dmetar” packages. Pooled prevalence estimates of clinical manifestations and outcomes were calculated using the inverse variance method with **a** Freeman-Tukey transformation to stabilize variances, particularly for proportions close to 0 or 1. A random-effects model was applied with the DerSimonian–Laird estimator (DL) for between-study variance (τ²), and the Jackson method (J) to estimate confidence intervals for τ².

The proportion of deaths and relapses attributable to each manifestation (i.e., the percentage of overall mortality or relapse explained by a given manifestation) was estimated using the same framework, with pooled proportions calculated via logit Freeman-Tukey transformation and a random-effects model with DL τ² estimation.

Case Fatality Rate (CFR) for mortality associated with abdominal signs, renal involvement, cardiac involvement, and central nervous system (CNS) involvement were calculated as the pooled proportion of manifestation-specific deaths among patients with that manifestation.

Correlation analyses were conducted to assess associations between clinical and epidemiological variables. Both Spearman’s rank correlation (ρ), a nonparametric method robust to monotonic but non-linear relationships, and Pearson’s correlation coefficient (r), which assumes linearity and approximate normality, were computed.

Results from individual studies, including prevalence for clinical manifestation, mortality and relapse rates were tabulated in summary tables in order to display the pooled prevalence and proportion estimates, as well as subgroup analysis (by age group). P-values were also included in these tables. All tables were constructed to clearly present study-level data and synthesized outcomes.

Heterogeneity was assessed using Cochran’s Q test and the Higgins I² statistic, with I² values interpreted as follows: 0–40% (low), 30–60% (moderate), 50–90% (substantial), and 75–100% (considerable heterogeneity. Statistical significance was set at *p* < 0.05 for all analyses.

Assessment of the presence of publication bias was achieved through the performance of Peter’s test and corresponding plots [15]. Finally, a leave-one-out sensitivity analysis was also conducted in order to investigate any potential differences in pooled prevalence of each manifestation.

### Assessment of certainty in the body of evidence

The overall certainty (or confidence) in the evidence for each outcome was qualitatively evaluated by considering the risk of bias of included studies, consistency of results, outcomes from Peter’s test, and the sensitivity analysis.

## Results

### Study characteristics

**Fig. 1:**
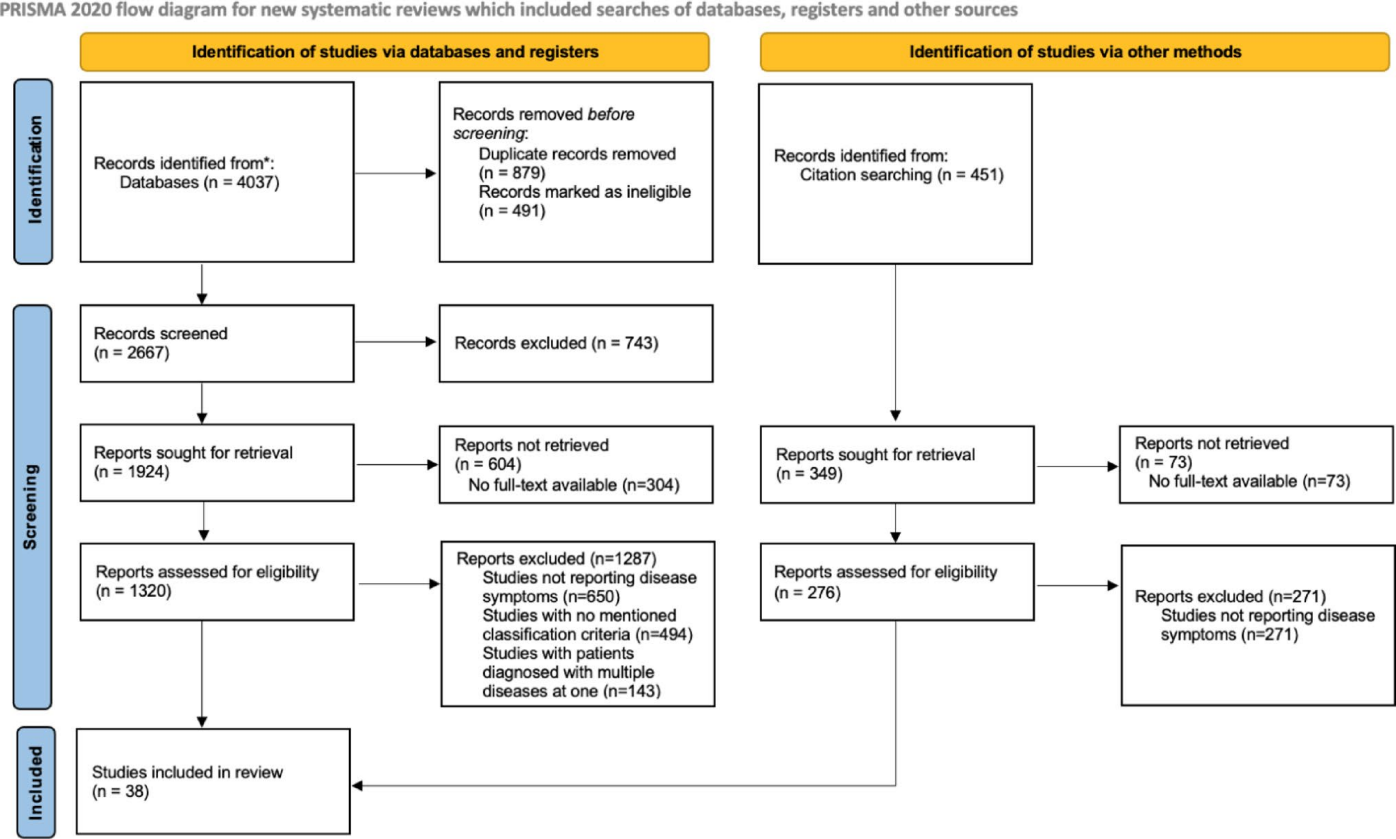
PRISMA flow diagram for study selection process

After the completion of our systematic search, 4488 studies were identified from databases and citation searching. Following the removal of duplicates and articles that were not retrieved, 1596 studies were assessed for eligibility. Finally, it was decided that 38 studies would be included in the systematic review (Fig. 1).

Among the included studies, 68.05 patients was the mean sample per study. Furthermore, 27 studies included Adult-based patient groups, while 14 assessed pediatric patients. 3 studies included both adult and pediatric groups. 9 studies belonged to the American population, 16 to the European, 11 to the Asian, while 2 studies were internationally conducted with patients from many regions. The study characteristics are presented in Table 1.

**Table 1:** Characteristics of the assessed studies, including year of publication, total patients, study type, patient age group, and risk of bias assessment

	Study names	Year	Area	Study design	Total patients	Age group	Risk of bias
1.	Guillevin [16]	1988	Europe	Retrospective cohort	165	Adult	Moderate risk
2.	Karadag [3]	2024	International	Retrospective cohort	358	Adult/Children	Moderate risk
3.	Pagnoux [4]	2010	Europe	Retrospective cohort	348	Adult	High risk
4.	Oner [5]	2016	Europe	Retrospective cohort	48	Adult	Low risk
5.	Sharma [6]	2016	Asia	Retrospective cohort	27	Adult	Moderate risk
6.	Bae [17]	2006	Asia	Retrospective cohort	27	Adult	Moderate risk
7.	Cohen [18]	1980	America	Retrospective cohort	53	Adult	High risk
8.	Fortin [19]	1995	America	Retrospective cohort	45	Adult	Moderate risk
9.	Agard [20]	2003	Europe	Retrospective cohort	36	Adult	Low risk
10.	Gupta [21]	1997	Asia	Retrospective case series	17	Adult	High risk
11.	Rohmer [22]	2023	Europe	Retrospective cohort	196	Adult	Low risk
12.	Campos [23]	2019	Europe	Retrospective cohort	31	Adult	Moderate risk
13.	M4rlin [24]	2015	Europe	Retrospective cohort	29	Children	Moderate risk
14.	Chen [25]	1989	Asia	Retrospective case series	20	Adult	High risk
15.	David [26]	1993	America	Retrospective case series	12	Children	High risk
16.	Kumar [27]	1995	Asia	Retrospective case series	10	Children	Moderate risk
17.	Ozen [28]	2004	Europe, America	Retrospective cohort	110	Children	Moderate risk
18.	Fathalla [29]	2005	America	Retrospective case series	4	Children	High risk
19.	Eleftheriou [30]	2013	Europe	Retrospective cohort	69	Children	Low risk
20.	Daoud [31]	1996	America	Retrospective cohort	79	Adult	High risk
21.	Selga [32]	2006	Europe	Retrospective case series	10	Adult	Low risk
22.	Leib [33]	1979	America	Retrospective cohort	64	Adult	Moderate risk
23.	Travers [34]	1979	Europe	Retrospective case series	17	Adult	High risk
24.	Sonmez [35]	2018	Asia	Retrospective cohort	133	Adult/Children	Moderate risk
25.	Guillevin [36]	2005	Europe	Retrospective cohort	115	Adult	Moderate risk
26.	Lai [37]	2021	Asia	Retrospective cohort	145	Adult	Moderate risk
27.	Levine [38]	2002	America	Retrospective case series	24	Adult	Moderate risk
28.	Lee [39]	2021	Asia	Retrospective case series	9	Children	High risk
29.	Ribi [40]	2010	Europe	Retrospective cohort	58	Adult	Low risk
30.	Jelusic [41]	2012	Europe	Retrospective case series	9	Children	Moderate risk
31.	Erden [42]	2017	Asia	Retrospective cohort	37	Adults/Children	Moderate risk
32.	Gayraud [43]	2001	Europe	Retrospective cohort	159	Adult	Moderate risk
33.	Ettlinger [44]	1979	America	Retrospective case series	9	Children	High risk
34.	Magilavy [45]	1977	America	Retrospective case series	9	Children	Low risk
35.	Mondal [46]	2013	Asia	Retrospective case series	15	Adult	Moderate risk
36.	Tekgoz [47]	2021	Asia	Retrospective case series	19	Children	Low risk
37.	Samson [48]	2014	Europe	Retrospective cohort	57	Adult	Low risk
38.	Kint [49]	2014	Europe	Retrospective case series	13	Adult	High risk

### Constitutional symptoms

#### Fever

The pooled estimated prevalence of fever was calculated after it was corrected through the trim and fill method, according to the Peter’s test results. The prevalence was found to be 61.2% with 95% CI: 0.53; 0.68. According to the subgroup analysis, prevalence of this manifestation was 51.5% in adults with 95% CI: 0.44; 0.59, while in pediatric patients it was present in 82.0% with 95% CI: 0 0.68; 0.92. Thus, age group was a statistically significant moderator (p-value = 0.0003). For HBV related disease, the estimated prevalence was found to be 69.7% with 95% CI: 0.63; 0.75, while in non-HBV PAN it was reported in 56.4% with 95% CI: 0.49; 0.63. The result was statistically significant (p-value = 0.0089). Fever appeared to be more common in systemic PAN compared to the cutaneous type (60.1% vs. 44.7%) without, however, having a statistically significant difference. Regarding geographic area distribution, fever had a similar frequency in Europe, Asia and America (61.1% vs. 61.9% vs. 65.3%), without having a statistically significant difference.

**Fig. 2:**
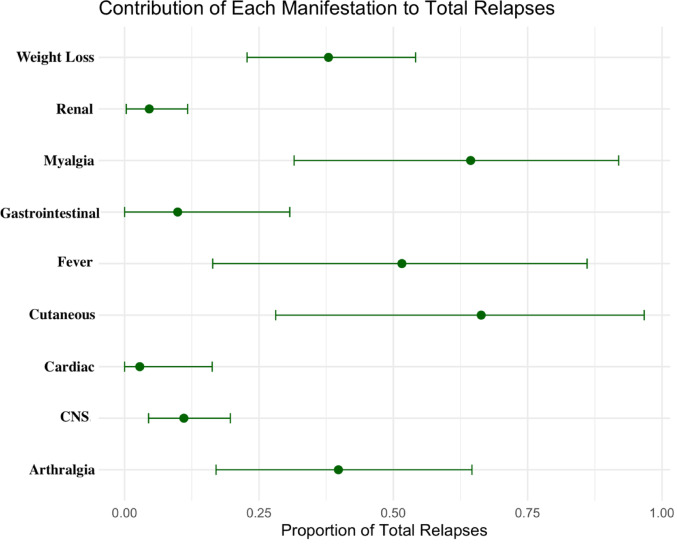
Analysis of clinical manifestations observed during disease relapse. The forest plot illustrates the contribution of specific clinical features to total relapse episodes

As far as disease outcomes are concerned, fever was present in 51.5% of the total relapses with 95% CI: 0.16; 0.86. Detailed statistics are presented in Tables 2 and 3, while relapse percentages are visualized in Fig. 2.

#### Weight loss

The pooled estimated prevalence of weight loss was calculated at 47.7% with 95% CI: 0.38; 0.56. According to the subgroup analysis, prevalence of this manifestation was 51.3% in adults with 95% CI: 0.41; 0.61, while in pediatric patients it was present in 39.1% with 95% CI: 0.20; 0.59. However, age group was not a statistically significant moderator. For HBV related disease, the estimated prevalence was found to be 87.8% with 95% CI: 0.76; 0.96, while in non-HBV PAN it was reported in 45.4% of cases with 95% CI: 0.07; 0.86. However, the result was not statistically significant. Weight loss was observed to be more common in systemic PAN compared to the cutaneous type (49.1% vs. 23.0%) while also having a statistically significant difference. Regarding geographic area distribution, weight loss was observed more often in Europe and America compared to Asia (59.3% and 63.1% vs. 39.0%), while showing a statistically significant difference.

As far as disease outcomes are concerned, weight loss was present in 37.9% of the total relapses with 95% CI: 0.22; 0.54 Detailed statistics are presented in Tables 2 and 3, while relapse percentages are visualized in Fig. 2.

Results for subgroups (random effects model):

### Musculoskeletal symptoms

#### Arthralgia

The pooled prevalence of arthralgia was estimated at 49.2% with 95% CI: 0.39; 0.55. According to the subgroup analysis, prevalence of this manifestation was 39.3% in adults with 95% CI: 0.32; 0.45, while in pediatric patients it was present in 73.9% with 95% CI: 0.64; 0.82. Thus, age group was a statistically significant moderator (p-value < 0.0001). The available data did not allow us to do a subgroup analysis for HBV-related PAN. Arthralgia was observed to be more common in cutaneous PAN compared to the systemic type (37.8% vs. 40.9%) without, however, having a statistically significant difference. Regarding geographic area distribution, arthralgia was observed less commonly in America (43.0%) compared to the other two areas (49.6% for Europe and 50.4% for Asia%), without having a statistically significant difference.

As far as disease outcomes are concerned, arthralgia was present in 39.7% of the total relapses with 95% CI0.17; 0.64. Detailed statistics are presented in Tables 2 and 3, while relapse percentages are visualized in Fig. 2.

#### Myalgia

The pooled estimated prevalence of myalgia was calculated at 54.3% with 95% CI: 0.47; 0.61. According to the subgroup analysis, prevalence of this manifestation was 53.2% in adults with 95% CI: 0.45; 0.60, while in pediatric patients it was present in 56.4% with 95% CI: 0.40; 0.71. However, age group was not a statistically significant moderator. For HBV related disease, the estimated prevalence was found to be 56.5% with 95% CI: 0.40; 0.72, while in non-HBV PAN it was present in 46.1% with 95% CI: 0.17; 0.76. However, the result was not statistically significant. Myalgia appeared to be more common in systemic PAN compared to the cutaneous type (57.4% vs. 29.3%) while also having a statistically significant difference. Regarding geographic area distribution, myalgia had a higher frequency in European populations (59.9%) and lower in American (44.5%), with Asian being in the middle (54.6%) without, however, having a statistically significant difference.

As far as disease outcomes are concerned, myalgia was present in 64.3% of the total relapses with 95% CI: 0.31; 0.91. Detailed statistics are presented in Tables 2 and 3, while relapse percentages are visualized in Fig. 2.

### Cutaneous involvement

The pooled estimated prevalence of cutaneous involvement was calculated after it was corrected through the trim and fill method, according to the Peter’s test results. The prevalence was found to be 64.8% with 95% CI: 0.55; 0.73. According to the subgroup analysis, prevalence of this manifestation was 57.8% in adults with 95% CI: 0.47; 0.67, while in pediatric patients it was present in 77.5% with 95% CI: 0.59; 0.92. However, age group was not a statistically significant moderator. For HBV related disease, the estimated prevalence was found to be 31.5% with 95% CI: 0.25; 0.37, while in non-HBV PAN it was present in 60.6% with 95% CI: 0.43; 0.76. The result was statistically significant (p-value = 0.0029). As expected, cutaneous signs were far more common in cutaneous PAN, in comparison to the systemic type (100% vs. 54.6%), creating a highly statistically significant difference. Regarding geographic area distribution, cutaneous signs had a higher frequency in Asian populations (72.9%) and lower in European (54.2%), with American being in the middle (62.7%) without, however, having a statistically significant difference.

As far as disease outcomes are concerned, cutaneous involvement was present in 66.3% of the total relapses with 95% CI: 0.28; 0.96. Detailed statistics are presented in Tables 2 and 3, while relapse percentages are visualized in Fig. 2.

### Hypertension

The corrected estimated pooled prevalence of hypertension based on trim and fill method was calculated at 30.6% with 95% CI: 0.23; 0.37. According to the subgroup analysis, prevalence of this manifestation was 34.5% in adults with 95% CI: 0.25; 0.43 while in pediatric patients it was present in 22.6% with 95% CI: 0.13; 0.33 Thus, age group was not a statistically significant moderator. For HBV related disease, prevalence was found to be 27.2% with 95% CI: 0 0.09; 0.49, while in non-HBV PAN it was present in 13.5% with 95% CI: 0.03; 0.28. However, the result was not statistically significant. Hypertension was observed to be more common in systemic PAN compared to the cutaneous type (34.8% vs. 5.6%) while also having a statistically significant difference. Regarding geographic area distribution, hypertension had a much lower frequency in Europe (21.3%) compared to Asia and America that had double that percentage and thus showing a statistically significant difference.

The available data did not allow us to calculate the relapses that hypertension was part of. Detailed statistics are presented in Tables 2 and 3.

### Gastrointestinal involvement

The pooled estimated prevalence of gastrointestinal (GI) involvement was calculated at 37.6% with 95% CI: 0.31; 0.43. According to the subgroup analysis, prevalence of this manifestation was 36.5% in adults with 95% CI: 0.30; 0.43, while in pediatric patients it was present in 40.0% with 95% CI: 0.26; 0.54. However, age group was not a statistically significant moderator. For HBV related disease, the estimated prevalence was found to be 41.5% with 95% CI: 0.25; 0.58, while in non-HBV PAN it was present in 26.7% with 95% CI: 0.14; 0.40. However, the result was not statistically significant. No cases of gastrointestinal involvement were recorded for patients with cutaneous PAN. Regarding geographic area distribution, gastrointestinal symptoms were notably more common in America (57.1%) in comparison to Asia and Europe (~ 29–36%) without, however, having a statistically significant difference.

**Fig. 3:**
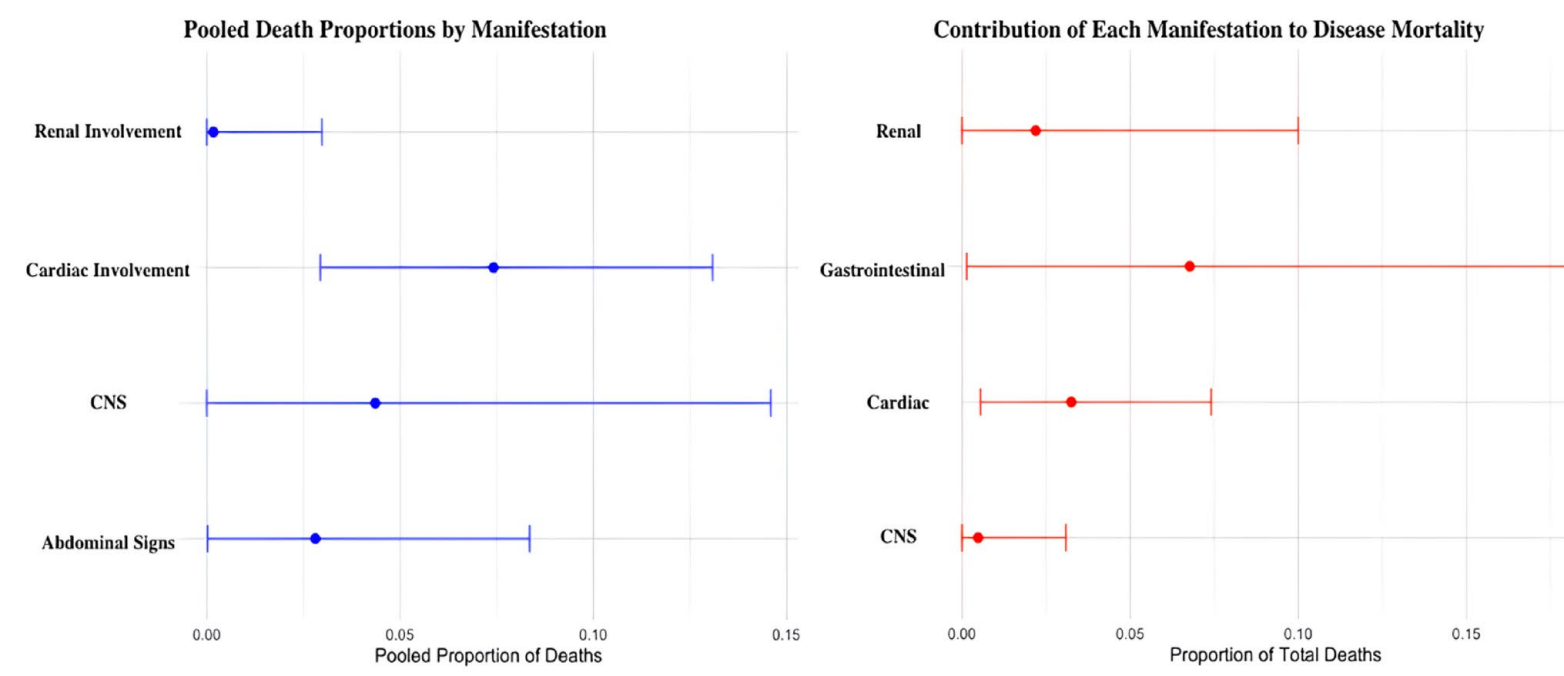
Mortality analysis by clinical manifestation. (Left) Forest plot displaying the pooled Case Fatality Rate (CFR) for specific clinical presentations. This represents the risk of death among patients affected by each manifestation. (Right) The distribution of total reported deaths attributed to each clinical manifestation, illustrating the contribution of each phenotype to the overall mortality burden

As far as disease outcomes are concerned, gastrointestinal involvement was estimated to be present in 9.8% of the total relapses with 95% CI: 0.00; 0.30. The case Fatality Rate (CFR) for such manifestation was calculated at 2.8% with 95% CI: 0.00; 0.08. Finally, gastrointestinal involvement accounted for 6.7% of Polyarteritis Nodosa deaths with 95% CI: 0.01; 0.18. Detailed statistics are presented in Tables 2 and 3, while CFR, relapse and mortality percentages are visualized in Figs. 2 and 3.

### Cardiac involvement

The corrected estimated pooled prevalence of cardiac involvement based on trim and fill method was calculated at 14.5% with 95% CI: 0.09; 0.20. According to the subgroup analysis, prevalence of this manifestation was 13.5% in adults with 95% CI: 0.07; 0.20, while in pediatric patients it was present in 18.1% with 95% CI: 0.05; 0.35. However, age group was not a statistically significant moderator. For HBV related disease, the estimated prevalence was found to be 18.9% with 95% CI: 0.12; 0.26 while in non-HBV PAN it was present in 11.8% with 95% CI: 0.01; 0.27. However, the result was not statistically significant. No cases of cardiac involvement were recorded for patients with cutaneous PAN. Regarding geographic area distribution, cardiac involvement followed roughly the same pattern as gastrointestinal involvement, with America having the highest prevalence (47.5%), while Asia and Europe had almost 5 times smaller frequency (~ 5–15%).

As far as disease outcomes are concerned, cardiac involvement was present in 2.8% of the total relapses with 95% CI: 0.00; 0.16. The CFR for such manifestation was calculated at 7.4% with 95% CI 0.02; 0.13. Finally, cardiac involvement was estimated to account for 3.2% of total disease deaths with 95% CI: 0.00; 0.07. Detailed statistics are presented in Tables 2 and 3, while CFR, relapse and mortality percentages are visualized in Figs. 2 and 3.

### Neurological symptoms

#### Central nervous system involvement (CNS)

The pooled prevalence of CNS involvement was estimated at 12.7% with 95% CI: 0.09; 0.16. According to the subgroup analysis, prevalence of this manifestation was 11.8% in adults with 95% CI: 0.08; 0.15, while in pediatric patients it was present in 15.7% with 95% CI: 0.09; 0.23. However, age group was not a statistically significant moderator. For HBV related disease, the estimated prevalence was found to be 3.9% with 95% CI: 0.00; 0.09 while in non-HBV PAN it was present in 13.0% with 95% CI: 0.02; 0.28. However, the result was not statistically significant. No cases of CNS involvement were recorded for patients with cutaneous PAN. Regarding geographic area distribution, CNS involvement is more commonly reported in American patients (18.7%), while Asia had the lowest prevalence (7.1%). However, these findings did not show a statistically significant difference.

As far as disease outcomes are concerned, CNS involvement was present in 11.0% of the total relapses with 95% CI: 0.04; 0.19. The CFR for this manifestation was calculated at 4.3% with 95% CI: 0.00; 0.14. Finally, CNS involvement accounted for around 0.4% of disease deaths with 95% CI: 0.00; 0.03. Detailed statistics are presented in Tables 2 and 3, while CFR, relapse and mortality percentages are visualized in Figs. 2 and 3.

#### Peripheral neuropathy

The pooled estimated prevalence of peripheral neuropathy involvement was calculated at 41.6% with 95% CI: 0.31; 0.52. According to the subgroup analysis, prevalence of this manifestation was 51.3% in adults with 95% CI: 0.41; 0.61, while in pediatric patients it was present in 12.6% with 95% CI: 0.03; 0.24. Thus, age group was a statistically significant moderator (p-value < 0.0001). For HBV related disease, prevalence was found to be 73.8% with 95% CI: 0.22; 1.00 while in non-HBV PAN it was found to be present in 68.9% with 95% CI: 0.62; 0.74. However, the result was not statistically significant. Peripheral Neuropathy was found to be more common in systemic PAN compared to the cutaneous type (42.2% vs. 19.8%) without, however, having a statistically significant difference. Regarding geographic area distribution, peripheral neuropathy had a fairly similar frequency in Europe, Asia and America (42.2% vs. 48.8% vs. 40.0%), without having a statistically significant difference.

The available data did not allow us to calculate the relapses that peripheral neuropathy was part of. Detailed statistics are presented in Tables 2 and 3, while relapse percentages are visualized in Fig. 2.

### Renal involvement

The pooled estimated prevalence of Renal involvement was calculated at 31.0% with 95% CI: 0.24; 0.38. According to the subgroup analysis, prevalence of this manifestation was 34.0% in adults with 95% CI: 0.26; 0.42, while in pediatric patients it was present in 25.0% with 95% CI: 0.12; 0.39. However, age group was not a statistically significant moderator. For HBV related disease, prevalence was estimated to be 52.2% with 95% CI: 0.31; 0.73 while in non-HBV PAN it was present in 32.9% with 95% CI: 0.09; 0.61. However, the result was not statistically significant. No cases of renal involvement were recorded for patients with cutaneous PAN. Regarding geographic area distribution, American individuals had the most renal manifestations (39.6%), while Asia had almost half of that prevalence at 21.4%, without having a statistically significant difference.

As far as disease outcomes are concerned, renal involvement was present in 4.5% of the total relapses with 95% CI: 0.00; 0.11. The CFR for this manifestation was calculated at 0.17% with 95% CI: 0.00; 0.02. Finally, renal involvement is accounted for 2.19% of disease deaths, with 95% CI: 0.00; 0.10. Detailed statistics are presented in Tables 2 and 3, while CFR, relapse and mortality percentages are visualized in Figs. 2 and 3.

### Mortality

The pooled prevalence of overall mortality was estimated at 13.5% with 95% CI: 0.08; 0.19. According to the subgroup analysis, death prevalence was 18.7% in adults with 95% CI: 0.13; 0.24, while 3.2% of pediatric patients died with 95% CI: 0 0.00; 0.13. Age group was a statistically significant moderator (p-value = 0.0155). For HBV related disease, prevalence was found to be 31.9% with 95% CI: 0.25; 0.38 while in non-HBV PAN it was present in 11.7% with 95% CI: 0.02; 0.27. Notably, the result was statistically significant (p-value = 0.0139). No deaths were reported in patients with cutaneous PAN compared to individuals with the systemic type. Regarding geographic distribution, statistically significant differences were observed as America had the highest lethality at 23.2%, while Asia had the lowest at 4.6%.

The Pearson correlation analysis between disease duration and mortality number was calculated at 0.49 with 95% CI: -0.19;0.85. As far as duration of follow-up is concerned, the correlation was calculated at -0.01 with 95% CI: -0.52;0.50, with no statistically significant effect. Detailed statistics are presented in Tables 2 and 3.

### Relapse

The pooled estimated prevalence of overall relapse was calculated at 28.2% with 95% CI: 0.20; 0.36. According to the subgroup analysis, relapse prevalence was 28.3% in adults with 95% CI: 0 0.18; 0.38, while 26.6% of pediatric patients relapsed with 95% CI: 0.14; 0.45. However, age group was not a statistically significant moderator. For HBV related disease, prevalence was found to be 6.3% with 95% CI: 0.03; 0.09 while in non-HBV PAN it was present in 19.1% with 95% CI: 0.09; 0.30. The result was statistically significant (p-value = 0.0336). Relapse was found to be more common in cutaneous PAN compared to the systemic type (34.1% vs. 26.0%) without, however, having a statistically significant difference. Geographic distribution analysis showed that America had the lowest rate of relapse at 11.9%, while on the contrary, Europe and Asia had more than double that percentage (30.3% and 25.2%). These differences were not, however, statistically significant.

The Pearson correlation analysis between disease duration and relapse number was calculated at 0.62 with 95% CI: -0.25;0.93. This result was not statistically significant. As far as duration of follow-up is concerned, the correlation was calculated at 0.16 with 95% CI: -0.42;0.65, with no statistically significant effect. Detailed statistics are presented in Tables 2 and 3.

### Remission

The pooled estimated prevalence of overall remission was calculated at 65.1% with 95% CI: 0.51; 0.77. According to the subgroup analysis, remission prevalence was 67.9% in adults with 95% CI: 0.51; 0.82, while 60.0% of pediatric patients achieved remission with 95% CI: 0.34; 0.83. However, age group was not a statistically significant moderator. For HBV related disease, prevalence was found to be 65.0% with 95% CI: 0.55; 0.74 while in non-HBV PAN it was present in 71.2% with 95% CI: 0.30; 0.98. The result was not statistically significant. Detailed Results can be found in Supplementary File 1. Remission occurred more often in systemic PAN compared to the cutaneous type (65.2% vs. 58.3%) without, however, having a statistically significant difference. The geographic analysis followed the same pattern as the relapse findings, with America having significantly lower remission at 20.3%, while Europe and Asia demonstrated almost quadruple that rate (77.7% and 83.4%).

Correlation analysis for disease duration was calculated at -0.04 with 95% CI: -0.89;0.87 and a non-significant p-value. For follow-up duration the result was 0.17 with 95% CI: 0.47;0.70. Detailed statistics are presented in Tables 2 and 3.

The detailed results of the HBV, systemic/cutaneous, and geographic area subgroup analyses can be found in Supplementary File 1.

**Table 2:** Prevalence results for each disease manifestation, as well as subgroup comparisons between adult and pediatric patients. Asterisks signify statistical significance.

Parameters	Prevalence (%)	Prevalence in adults (%)	Prevalence in pediatric patients (%)	*p*-value
Fever	61.2	51.5	82.0	0.0003*
Weight loss	47.7	51.3	39.1	0.2979
Myalgia	54.3	53.2	56.4	0.7251
Cutaneous involvement	64.8	57.8	77.5	0.0726
Arthralgia	49.2	39.3	73.9	< 0.0001*
Hypertension	30.6	34.5	22.6	0.0296*
Gastrointestinal involvement	37.6	36.5	40.0	0.6156
Cardiac involvement	14.5	13.7	18.1	0.4891
CNS involvement	12.7	11.8	15.7	0.1611
Peripheral neuropathy	41.6	51.3	12.6	< 0.0001*
Renal involvement	31.0	34.0	25.0	0.3005
Mortality	13.5	18.7	3.2	0.0155*
Relapse	28.2	28.3	25.6	0.8894
Remission	65.1	67.7	60.0	0.5698

**Table 3:** Clinical severity and relapse burden by manifestation

	Case fatality rate (%)	Responsible deaths (%)	Relapse contribution (%)
Fever	N/A	N/A	51.5
Weight loss	N/A	N/A	37.9
Myalgia	N/A	N/A	64.3
Cutaneous involvement	N/A	N/A	66.3
Arthralgia	N/A	N/A	39.7
Hypertension	N/A	N/A	N/A
Gastrointestinal involvement	2.8	6.7	9.8
Cardiac involvement	7.4	3.2	2.8
CNS involvement	4.3	0.4	11.0
Peripheral neuropathy	N/A	N/A	N/A
Renal involvement	0.1	2.1	4.5

This table summarizes the case fatality rate (CFR), the proportionate contribution of each manifestation to relapse episodes, and the distribution of total reported deaths

### Sensitivity analysis

A leave-one-out sensitivity analysis showed that no individual study unduly influenced the pooled prevalence estimates for any type. All manifestation pooled estimates were inside the expected ranges, while heterogeneity remained roughly constant throughout the analysis. Precisely, fever prevalence varied between 50.4 and 53.6%, while maintaining high heterogeneity (I^2^≅ = 90%). For weight loss, it varied between 46.1 and 50.1 with steady heterogeneity (I^2^≅93%). For myalgia 52.8–55.4% with I^2^≅84%. For Cutaneous involvement it was between 54.3 and 58.0% with I^2^≅92%. For arthralgia it was between 44.5 and 48.3% with I^2^≅79%. For hypertension between 17.5 and 21.7% with I^2^≅92%. For gastrointestinal involvement, it was between 32.8 and 36.9% with I^2^≅60%. For cardiac involvement, 9.3–14.8% with I^2^≅85%. For peripheral neuropathy, 39.6–44.7% with I^2^≅96%. For renal involvement, 27.3–34.9% with I^2^≅59%.

### Risk of bias assessment

Each study that was included in this analysis, was assessed according to the Quality in Prognostic Studies (QUIPS) Tool. The detailed bias assessment of the methodological quality of the studies is presented in Supplementary File 2. Additionally, Peter’s test for funnel plot asymmetry showed some evidence for fever, hypertension, cardiac involvement, and cutaneous signs. The prevalences were corrected using the trim and fill method [Supplementary File 3]. Finally, a subgroup analysis based on the different levels of risk of bias was conducted for each manifestation. All of the results came out as nonsignificant, highlighting the robustness of the results [Supplementary File 4].

## Discussion

This systematic review with meta-analysis aimed to investigate the clinical profile of Polyarteritis Nodosa, evaluating the frequency of manifestations, their clinical gravity, as well as the correlation and impact of several patient and disease characteristics on the patient prognosis. To the best of our knowledge, this represents one of the most comprehensive meta-analytic assessments of the clinical profile of this disease to date.

A notable finding in our analysis was the substantial between-study heterogeneity (> 80–90%) observed across most pooled prevalence estimates. This variability is methodologically significant and necessitates a cautious interpretation of the quantitative results. The variance in prevalence estimates is likely driven by a combination of factors, including differences in diagnostic criteria, diverse patient populations, and inclusion of different disease phenotypes. While this broad approach provides a comprehensive overview of the literature, it inevitably introduces substantial variance. Consequently, the pooled prevalence values presented here should be interpreted as weighted summary estimates of the existing literature rather than precise predictive probabilities for individual patients.

Keeping this limitation in mind, the results of this meta-analysis estimate that the most common manifestation of Polyarteritis Nodosa is cutaneous involvement, which can be observed in multiple forms, such as Nodules or Livedo reticularis. The least common clinical symptom appeared to be Cardiac involvement, as well as the involvement of the Central Nervous System, which has been reported in roughly similar rates among the total patients (~ 14%). Regarding disease outcomes, the pooled data suggest an overall mortality rate of approximately 15%, while in terms of clinical course, remission was estimated at ~ 65%, and relapses in ~ 30% of the aggregated patient population. As far as the Patient age difference is concerned, statistically significant results were found regarding the clinical manifestations of fever, arthralgia, hypertension, and peripheral neuropathy. Precisely, fever and arthralgia were predominantly found in pediatric patients, while hypertension and peripheral neuropathy were observed in the majority of cases in the adults. It should also be noted that the adult patient group presented with a mortality rate approximately 6 times larger than that of the pediatric group, indicating a significant disparity regarding the lethality of the disease in different age groups. Regarding the association with HBV, several general trends emerge. HBV-related polyarteritis nodosa is associated with a higher prevalence of severe and potentially life-threatening manifestations, including renal involvement, gastrointestinal symptoms, and cardiac involvement. Consequently, this subgroup demonstrates a significantly higher mortality rate compared with non-HBV-related polyarteritis nodosa (~ 32% vs. ~ 12%). In contrast, the prevalence of manifestations that are more commonly associated with disease relapse appears to be broadly similar between HBV-related and non-HBV-related cases. The highlight in this category of findings is the significantly higher prevalence of cutaneous involvement in non-HBV PAN, a symptom that has the highest correlation to relapse. Therefore, the observation that non-HBV PAN is associated with a higher rate of relapse aligns with established clinical expectations.

The systemic/cutaneous subgroup analysis reported that systemic PAN is a more severe form of the disease, compared to the cutaneous type. Apart from cutaneous signs and arthralgia, systemic polyarteritis nodosa had a notably higher association with all the other manifestations, including the more clinically lethal ones. Hence, higher mortality rates were reported in individuals with systemic PAN. However, relapse was more frequent in cutaneous PAN. This finding is expected, as cutaneous manifestations were the main contributors to relapse in the overall analysis, and by definition, cutaneous PAN is characterized by predominant skin involvement. As far as the geographic analysis is concerned, the main trends identified were a consistent association between a higher prevalence of clinically severe symptoms and studies conducted in American populations. On the contrary, Asian and European patients presented more often with either less severe signs or more relapse-associated symptoms, such as myalgia. Thus, American patients experienced a higher mortality rate and lower remission, while Asian and European individuals had more relapses during their recovery period. These geographic discrepancies further underscore the heterogeneity of the underlying data, suggesting that regional differences in genetic background, environmental factors, or healthcare referral patterns may influence reported disease phenotypes.

Overall, in terms of clinical severity, HBV-associated PAN, systemic disease, as well as American patient population are the three main factors that are correlated to more deaths and a more severe clinical picture.

In terms of manifestation gravity, cardiac involvement was found to be associated with the highest risk for patients, with a case fatality rate of approximately 7%. On the other hand, despite the high clinical risk for patients with Cardiac involvement and CNS participation, the low prevalence of such manifestations makes them a less constant threat for patients with Polyarteritis Nodosa. This can also be deduced from the proportion of accounted deaths by these highly dangerous manifestations, where they make up less than 4% of the total mortality combined. On the other hand, despite the fact that gastrointestinal and renal involvement do not present the same lethality as cardiac or CNS manifestations, their increased frequency translates into a higher absolute mortality burden. Therefore, it is clinically important that patients who present with any cardiac or CNS involvement should be followed closely and administered timely and appropriate therapy in order to avoid the high lethality that is associated with them. De Virgilio et al. review also states that involvement of a critical organ necessitates the adjustment of therapy, and specifically cyclophosphamide along with corticosteroids in these cases [50]. However, our findings should also act as a warning not to underestimate the clinical severity of patients who present with lower CFR system involvement, like gastrointestinal or renal manifestations. Thus, aggressive therapy, such as the one mentioned above, can be beneficial in these patients as well, while noting that the dose of cyclophosphamide should be adjusted in case the patients develop renal failure [51].

As far as relapses are concerned, a substantial majority of the patients who experienced at least one relapse presented with cutaneous signs, as well as myalgia. Fever could also be observed in slightly more than half of the total relapse cases. Therefore, patients who present with such clinical features have a notably higher association with relapse along the recovery path. Other variables, such as disease duration and follow-up period were not found to have a remarkable correlation with mortality, relapses, or remission. Hence, even though manifestations like cutaneous symptoms or myalgia do not contribute significantly to the overall mortality rate, appropriate therapy, typically less aggressive, is also important as these symptoms are among the top responsible features for relapse. Papachristodoulou et al. concur that cutaneous manifestations and cutaneous PAN are associated with an increased risk of relapse, and state that long term low doses of corticosteroids should be administered to prevent them [52].

Even though there is a lack of systematic reviews and meta-analyses regarding the manifestation prevalence as well as risk and prognostic factors regarding polyarteritis nodosa, several narrative reviews have been published. Rodriguez et al. study [53] supports the idea that fever, weight loss, myalgia and arthralgia are present in 31–69%, 16–69%, 30–59%, and 44–59% respectively. All of our findings lay within those ranges, with the majority of them belonging to the upper part of Rodriguez’s estimation. Furthermore, the same assessment can be applied to gastrointestinal involvement, renal involvement, cardiac involvement, and CNS involvement, all of which fall inside the reported percentages reported by Rodriguez (14–44%, 8–66%, 4–30%, 2–28% respectively). Differences can be observed regarding the prevalence of peripheral neuropathy and cutaneous involvement. While Rodriguez review reports a 74% frequency for neuropathy and 28–58% for cutaneous signs, our meta-analysis suggests a moderate estimate of approximately 40% for the former and slightly higher (~ 65%) for the latter. The aforementioned study also states that recent findings contradict previous data that estimated the relapse rate at around 10%. This aligns with our findings, which indicate a higher estimated relapse burden, likely closer to ~ 30%.

A more recent review by De Virgilio states that cutaneous involvement and peripheral neuropathy are the most common manifestations of polyarteritis nodosa [50]. While skin association was indeed our most common manifestation, the same cannot be said about peripheral neuropathy, which ranked lower among the most common clinical symptoms. Moreover, the same study supports that gastrointestinal tract symptoms are the most serious expression of the disease. Even though our CFR finding cannot support that, the fact that GI manifestations are responsible for a bigger proportion of total deaths compared to the rest of the symptoms means that our results align with this conclusion.

The most recent review regarding polyarteritis nodosa, by Wolff et al., also gives some estimates regarding symptom frequency [1]. All our findings fall within their reported ranges. However, it should be noted that CNS involvement is estimated to occur in 2–10% of patients. On the other hand, our meta-analysis points to a slightly increased prevalence of around 15%, while notably this percentage becomes slightly greater in pediatric patients.

Another recent article by Mohankumar et al. compared and presented the clinical features of Kawasaki disease and Polyarteritis Nodosa [54]. Through this analysis, we found that both studies considered cardiac involvement a rare observation, while the multisystem involvement of gastrointestinal and renal symptoms were among the highest in frequency. Another review by Misra et al. explores the cardiac involvement in different systemic vasculitis. As far as polyarteritis nodosa is concerned, the study emphasizes the increased risk of mortality for patients with the aforementioned system involvement [55]. Additionally, his findings also highlight the notable high prevalence of cardiac involvement in pediatric patients. Thus, all of the above findings were supported and expanded upon by our meta-analysis.

Furthermore, we believe that a more detailed comparison between the findings of this meta-analysis and the results from the international, multicentral study of Karadag et al. would further elucidate the key differences and the reasons behind them [3]. Both studies consistently demonstrate that constitutional symptoms, particularly fever and weight loss, are predominantly associated with systemic PAN, although the magnitude of this association was more modest in our pooled estimates.

Overall, the prevalence of most organ system involvements in systemic PAN was broadly comparable between the two datasets. Neurological manifestations, including central nervous system involvement and peripheral neuropathy, represented the most frequently affected systems in both analyses, albeit with a lower pooled prevalence in our study. A notable discrepancy was observed in renal involvement, which was substantially higher in the GLOBAL-PAN cohort (~ 48%) compared with our meta-analysis (~ 30%).

The greatest divergence between the two datasets emerged in subgroup analyses comparing pediatric and adult patients. Although both studies consistently confirmed a higher prevalence of fever among pediatric patients, associations with gastrointestinal, neurological, and renal involvement reported in the GLOBAL-PAN cohort were not replicated in our pooled estimates.

Several factors may explain these discrepancies. First, the GLOBAL-PAN cohort included a more geographically restricted population with a lower representation of Asian patients, whereas our analysis identified lower renal involvement in Asian cohorts, potentially inflating renal prevalence in the multicenter study. Second, differences in diagnostic strategy may have contributed, as the GLOBAL-PAN study relied predominantly on systematic imaging and histopathological confirmation rather than formal application of ACR or CHCC classification criteria. Third, hepatitis B–associated PAN cases were not included in the GLOBAL-PAN cohort, whereas such cases were captured in our analysis, potentially influencing organ-specific phenotypic distributions. Finally, both studies were concordant in demonstrating the relatively benign clinical course of cutaneous PAN, characterized by high remission rates and exceptionally low mortality.

## Strengths

This analysis is strengthened by a clearly defined and independently executed study selection and data extraction process, that can reduce the risk of bias. Additionally, the use of rigorous statistical methodology including extensive pooled prevalences, sub-group analyses, univariate analyses, p-value corrections, comprehensive risk of bias and publication bias assessments (QUIPS tool, Peters’ test, trim and fill method), and sensitivity analyses confirming the robustness of pooled estimates. The transparent use of validated tools and open-source statistical packages further enhances the reproducibility and reliability of the findings.

## Limitations

The findings of this systematic review and meta-analysis should be interpreted in light of several key limitations. A primary methodological constraint was the consistently high between-study heterogeneity (often exceeding 80–90%) observed across most pooled prevalence estimates. This variance likely reflects the broad inclusion criteria employed to capture the full clinical spectrum of the disease, including diverse patient populations and disease phenotypes. Consequently, the pooled estimates reported here should be viewed as weighted summary averages rather than precise predictive probabilities for individual patients. Regarding study quality, several studies were characterized as Moderate or High Risk according to the QUIPS et al. tool, while some of them have considerable chance of small study effect, which have been mitigated using a trim and fill method. The age distribution of studies was also imbalanced, with a predominance of data from adults and limited representation from pediatric-based studies, which may affect the generalizability of results. There were some limitations to clinical data reporting, as not all studies reported manifestation-caused deaths or relapses, resulting in a limited ability to explore the association between angiographic types and clinical patient symptoms.

This meta-analysis is based on aggregated observational data. Accordingly, these findings reflect reported associations rather than confirmed causality. Consequently, while certain manifestations were more frequently observed among patients who relapsed or died, definitive causal inferences regarding disease progression cannot be drawn without prospective, controlled data.

## Conclusion

In conclusion, this meta-analysis provides a comprehensive and systematic synthesis of the clinical manifestations, outcomes, and prognostic features of polyarteritis nodosa across diverse populations. Cutaneous involvement was identified as the most frequent manifestation, while CNS involvement was the least common but associated with high clinical risk. Cardiac and CNS manifestations, although rare, confer the greatest individual mortality risk, whereas more frequent gastrointestinal and renal involvement contribute to a higher absolute mortality burden. Pediatric patients predominantly exhibited fever and arthralgia, whereas adults more commonly presented with hypertension and peripheral neuropathy. Relapses were most often associated with cutaneous signs, myalgia, and fever, highlighting their prognostic significance. Overall, these findings offer valuable insight into the prevalence, severity, and prognostic implications of the disease manifestations. While this review cannot establish causal relationships or directly predict outcomes as the evidence is observational, heterogenous and indirect, the aggregated data may help physicians recognize common and high-risk manifestations and anticipate disease trajectories, potentially supporting more informed clinical decision-making.

## Future research directions

Based on our results, we recommend more population studies, exploring the prevalence and outcomes of Polyarteritis nodosa, in more clinically diverse patient groups (pediatric, HBV-associated, systemic vs. cutaneous) which were not as common. Additionally, studies with more detailed reporting of the clinical symptoms, as well as the direct cause of death or relapse of all patients is crucial, since it creates more opportunities to investigate and research characteristics and factors that impact the patients’ prognosis.

## Supplementary Information

Below is the link to the electronic supplementary material.

Supplementary Material 1

Supplementary Material 2

Supplementary Material 3

Supplementary Material 4

## Data Availability

No datasets were created.
