# Supplementary material for: Clinical manifestations, prognostic impact, and relapse in polyarteritis nodosa: a systematic review and meta-analysis
Source: Rheumatol Int. 2026 Feb 19;46(3):51. doi: 10.1007/s00296-026-06082-8 (PMC12920359; doi:10.1007/s00296-026-06082-8)
Supplement: Supplementary file 1 — Supplementary Material 1 [file 296_2026_6082_MOESM1_ESM.docx]

Table 1: Subgroup Analysis for HBV related and non-HBV PAN

| Parameters | Prevalence in HBV related PAN (%) | Prevalence in non-HBV PAN (%) | p-value |
| --- | --- | --- | --- |
| Fever | 69.7 | 56.4 | 0.0089* |
| Weight Loss | 87.8 | 45.4 | 0.0674 |
| Myalgia | 56.5 | 46.1 | 0.5750 |
| Cutaneous Involvement | 31.5 | 60.6 | 0.0029* |
| Arthralgia | - | - | - |
| Hypertension | 27.2 | 13.5 | 0.2049 |
| Gastrointestinal Involvement | 41.1 | 26.7 | 0.1554 |
| Cardiac Involvement | 18.9 | 11.8 | 0.2798 |
| CNS Involvement | 3.9 | 13.0 | 0.3140 |
| Peripheral Neuropathy | 73.8 | 68.9 | 0.8596 |
| Renal Involvement | 52.2 | 32.9 | 0.2852 |
| Mortality | 31.9 | 11.7 | 0.0139* |
| Relapse | 6.3 | 19.1 | 0.0336* |
| Remission | 65.0 | 71.2 | 0.7252 |

Table 2: Subgroup Analysis for Systemic and Cutaneous PAN

| Parameters | Prevalence in Systemic PAN (%) | Prevalence in Cutaneous PAN (%) | p-value |
| --- | --- | --- | --- |
| Fever | 60.1 | 44.7 | 0.2245 |
| Weight Loss | 49.1 | 23.0 | 0.0417* |
| Myalgia | 57.4 | 29.3 | 0.0003* |
| Cutaneous Involvement | 54.6 | 100.0 | <0.0001* |
| Arthralgia | 40.9 | 58.4 | 0.1312 |
| Hypertension | 34.8 | 5.6 | 0.0051* |
| Gastrointestinal Involvement | 41.1 | 0.0 | <0.0001* |
| Cardiac Involvement | 20.7 | 0.0 | <0.0001* |
| CNS Involvement | 15.1 | 0.0 | <0.0001* |
| Peripheral Neuropathy | 42.2 | 19.8 | 0.2030 |
| Renal Involvement | 31.2 | 0.0 | <0.0001* |
| Mortality | 14.5 | 0.0 | <0.0001* |
| Relapse | 26.0 | 34.1 | 0.2862 |
| Remission | 62.5 | 58.3 | 0.6916 |

Table 3: Subgroup Analysis for Geographic Areas

| Parameters | Prevalence in Europe (%) | Prevalence in Asia (%) | Prevalence in America (%) | p-value |
| --- | --- | --- | --- | --- |
| Fever | 61.1 | 61.9 | 65.3 | 0.9774 |
| Weight Loss | 59.3 | 39.0 | 63.1 | 0.0446* |
| Myalgia | 59.9 | 54.6 | 44.5 | 0.6392 |
| Cutaneous Involvement | 54.2 | 72.9 | 62.7 | 0.1773 |
| Arthralgia | 49.5 | 50.4 | 43.0 | 0.9581 |
| Hypertension | 21.3 | 39.5 | 42.0 | 0.0442* |
| Gastrointestinal Involvement | 36.6 | 29.3 | 57.1 | 0.2387 |
| Cardiac Involvement | 15.5 | 5.5 | 47.5 | 0.0107* |
| CNS Involvement | 11.7 | 7.1 | 18.7 | 0.4336 |
| Peripheral Neuropathy | 42.2 | 48.8 | 40.0 | 0.8800 |
| Renal Involvement | 30.5 | 21.4 | 39.6 | 0.4590 |
| Mortality | 17.3 | 4.1 | 23.2 | 0.0036* |
| Relapse | 30.3 | 25.2 | 11.9 | 0.6850 |
| Remission | 77.7 | 83.4 | 20.3 | <0.0001* |
