# Supplementary material for: Clinical manifestations, prognostic impact, and relapse in polyarteritis nodosa: a systematic review and meta-analysis
Source: Rheumatol Int. 2026 Feb 19;46(3):51. doi: 10.1007/s00296-026-06082-8 (PMC12920359; doi:10.1007/s00296-026-06082-8)
Supplement: Supplementary file 2 — Supplementary Material 2 [file 296_2026_6082_MOESM2_ESM.docx]

Table 1: Risk of Bias Assessment according to the Quality in Prognostic Studies (QUIPS) Tool

| Study Names | Study Participation | Study Attrition | Prognostic Factor Measurement | Outcome Measurement | Study Confounding | Statistical Analysis and Reporting |
| --- | --- | --- | --- | --- | --- | --- |
| Guillevin 1988 | Moderate Risk | Low Risk | Moderate Risk | Low Risk | High Risk | Moderate Risk |
| Karadag 2024 | Moderate Risk | Moderate Risk | Low Risk | Moderate Risk | Moderate Risk | Moderate Risk |
| Gagnoux | Moderate risk | High Risk | Low Risk | Moderate Risk | Moderate Risk | Moderate Risk |
| Oner | Moderate Risk | Low Risk | Low Risk | Low Risk | Moderate Risk | Low Risk |
| Sharma | Moderate Risk | Low Risk | Moderate Risk | Low Risk | Moderate Risk | Moderate Risk |
| Bae | Moderate Risk | Low Risk | Moderate Risk | Low Risk | Moderate Risk | Moderate Risk |
| Cohen | Moderate Risk | High Risk | Moderate Risk | Moderate Risk | High Risk | Moderate Risk |
| Fortin | Moderate Risk | Low Risk | Moderate Risk | Moderate Risk | Moderate Risk | Moderate Risk |
| Agard | Moderate Risk | Low Risk | Low Risk | Moderate Risk | Low Risk | Low Risk |
| Gupta | High Risk | Moderate Risk | Low Risk | Moderate Risk | High Risk | Moderate Risk |
| Rohmer | Low Risk | Low Risk | Moderate risk | Low Risk | Moderate Risk | Low Risk |
| Campos | Moderate Risk | Low Risk | Moderate Risk | Moderate Risk | High Risk | Moderate Risk |
| Merlin | Moderate Risk | Low Risk | Moderate Risk | Moderate Risk | Low Risk | Moderate Risk |
| Chen | Moderate Risk | Moderate Risk | Moderate Risk | Moderate Risk | High Risk | High Risk |
| David | Moderate Risk | Low Risk | Moderate Risk | High Risk | Moderate Risk | Moderate Risk |
| Kumar | Moderate Risk | Low Risk | Moderate Risk | Moderate Risk | Moderate Risk | Moderate Risk |
| Ozen | Moderate Risk | Low Risk | Low Risk | Moderate Risk | High Risk | Moderate Risk |
| Fathalla | High Risk | Low Risk | Moderate Risk | Moderate Risk | High Risk | High Risk |
| Eleftheriou | Moderate Risk | Low Risk | Low Risk | Low Risk | Moderate Risk | Low Risk |
| Daoud | Moderate Risk | Low Risk | Moderate Risk | Moderate Risk | High Risk | High Risk |
| Selga | Low Risk | Low Risk | Low Risk | Low Risk | Moderate Risk | Moderate Risk |
| Leib | Moderate Risk | Moderate Risk | Moderate Risk | Low Risk | High Risk | Moderate Risk |
| Travers | Moderate Risk | Low Risk risk | Moderate Risk | Low Risk | High Risk | High risk |
| Sonmez | Moderate Risk | Moderate Risk | Moderate Risk | Low Risk | High Risk | Moderate Risk |
| Guillevin | Moderate Risk | Low Risk | Moderate Risk | Low Risk | High Risk | Moderate Risk |
| Lai | Moderate Risk | Low Risk | Moderate Risk | Moderate Risk | High Risk | Moderate Risk |
| Levine | Moderate Risk | Low Risk | Moderate Risk | Low Risk | High Risk | Moderate Risk |
| Lee | High risk | Low Risk | Moderate Risk | Moderate Risk | High Risk | High Risk |
| Ribi | Low Risk | Low Risk | Moderate Risk | Low Risk | Moderate Risk | Moderate Risk |
| Jelusic | Moderate Risk | Low Risk | Moderate Risk | Moderate risk | High Risk | High Risk |
| Erden | Moderate Risk | Low Risk | Moderate Risk | Low Risk | High Risk | Moderate Risk |
| Gayraud | Low Risk | Low Risk | Moderate Risk | Moderate Risk | Moderate Risk | Moderate Risk |
| Ettlinger | High Risk | High Risk | Moderate Risk | High Risk | High risk | High Risk |
| Magilavy | Moderate Risk | Moderate Risk | Low Risk | Low Risk | Moderate Risk | Low Risk |
| Mondel | Moderate Risk | Moderate Risk | Moderate Risk | Low Risk | Moderate Risk | Low Risk |
| Tekgoz | Moderate Risk | Moderate Risk | Low Risk | Low Risk | Moderate Risk | Low Risk |
| Samson | Moderate Risk | Low Risk | Low Risk | Low Risk | Moderate Risk | Low Risk |
| Kint | Moderate Risk | Low Risk | Moderate Risk | Moderate risk | High Risk | High Risk |
