# Supplementary material for: Clinical manifestations, prognostic impact, and relapse in polyarteritis nodosa: a systematic review and meta-analysis
Source: Rheumatol Int. 2026 Feb 19;46(3):51. doi: 10.1007/s00296-026-06082-8 (PMC12920359; doi:10.1007/s00296-026-06082-8)

Peter’s Test Results

Fever

Model: mixed-effects meta-regression model

Predictor: standard error

Test for Funnel Plot Asymmetry: z = 2.6867, p = 0.0072

Limit Estimate (as sei -> 0): b = 0.7013 (CI: 0.5448, 0.8577)


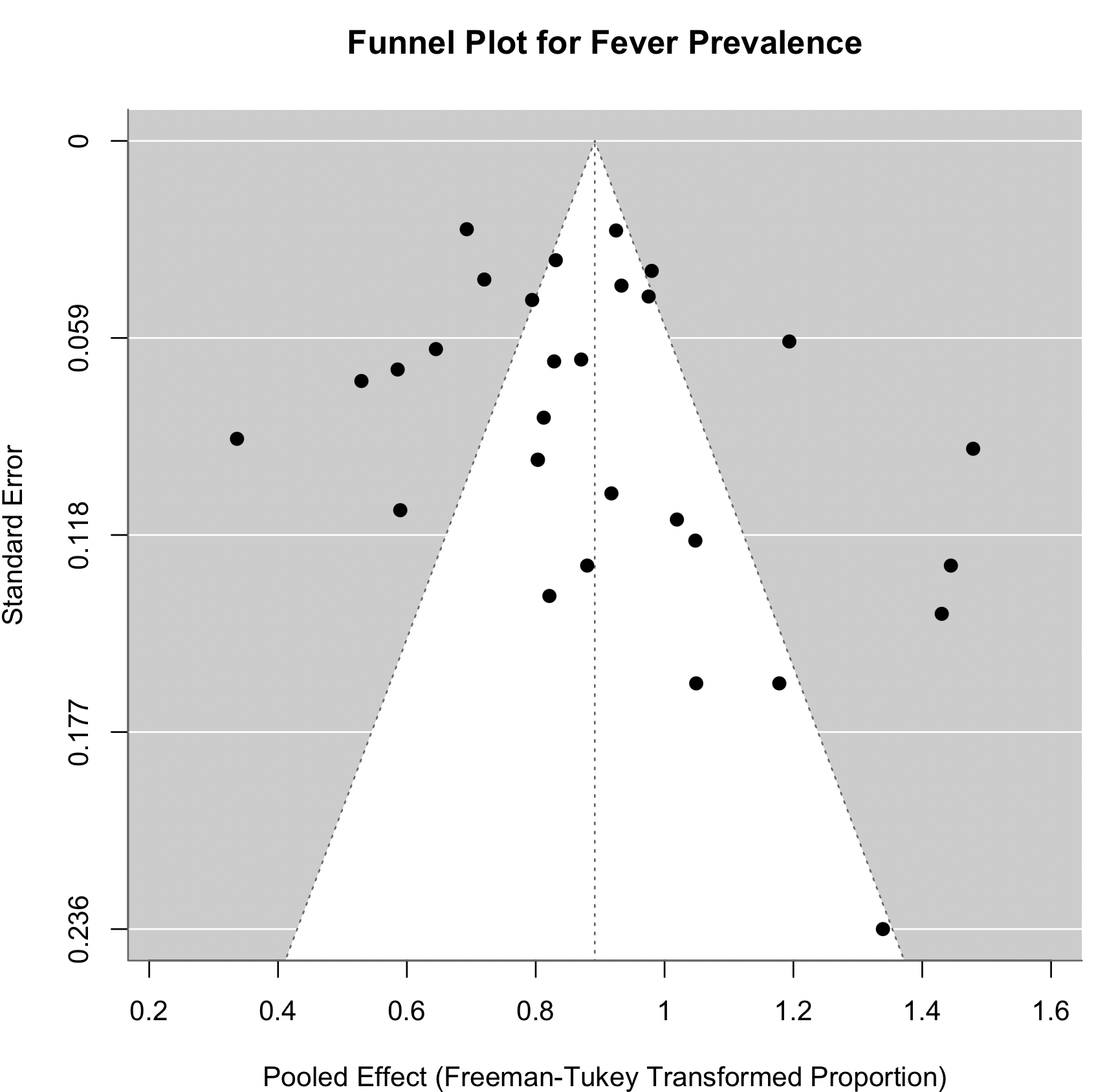


Weight Loss

Model: mixed-effects meta-regression model

Predictor: standard error

Test for Funnel Plot Asymmetry: z = -0.6516, p = 0.5147

Limit Estimate (as sei -> 0): b = 0.8340 (CI: 0.6204, 1.0475)


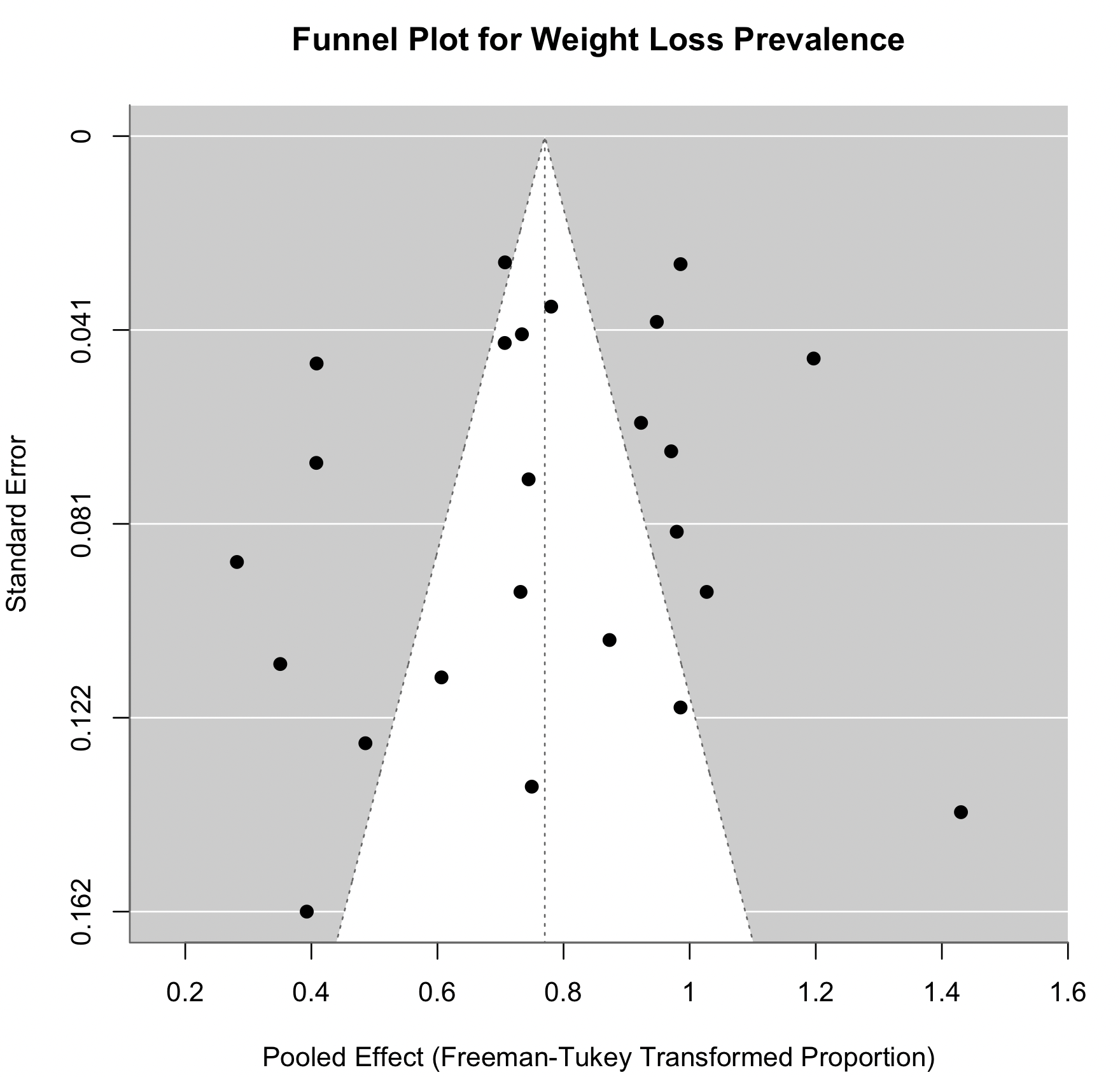


Myalgia

Model: mixed-effects meta-regression model

Predictor: standard error

Test for Funnel Plot Asymmetry: z = 1.4080, p = 0.1591

Limit Estimate (as sei -> 0): b = 0.7139 (CI: 0.5558, 0.8720)


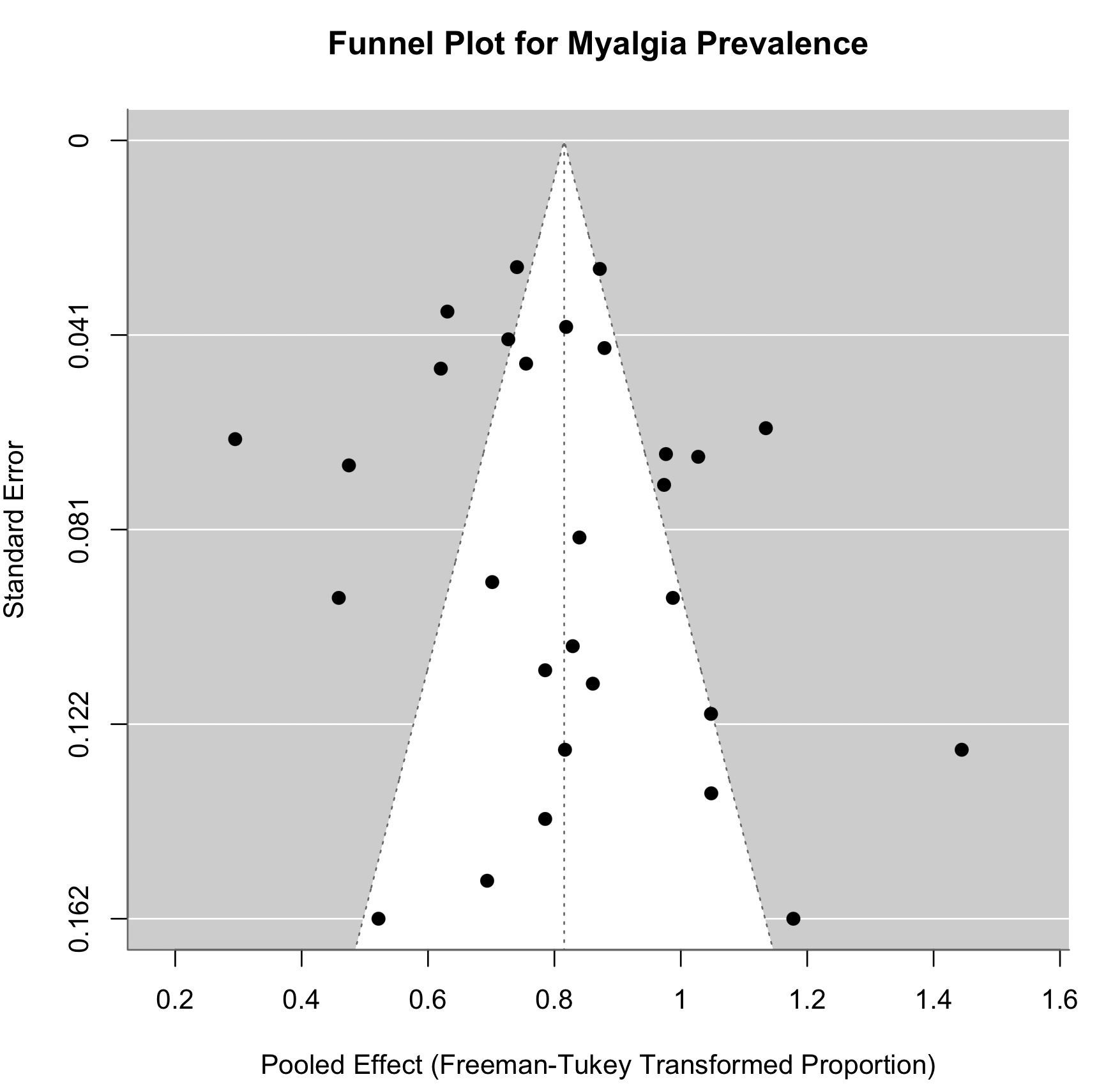


Cutaneous Signs

Model: mixed-effects meta-regression model

Predictor: standard error

Test for Funnel Plot Asymmetry: z = 1.9714, p = 0.0487

Limit Estimate (as sei -> 0): b = 0.7485 (CI: 0.5576, 0.9395)


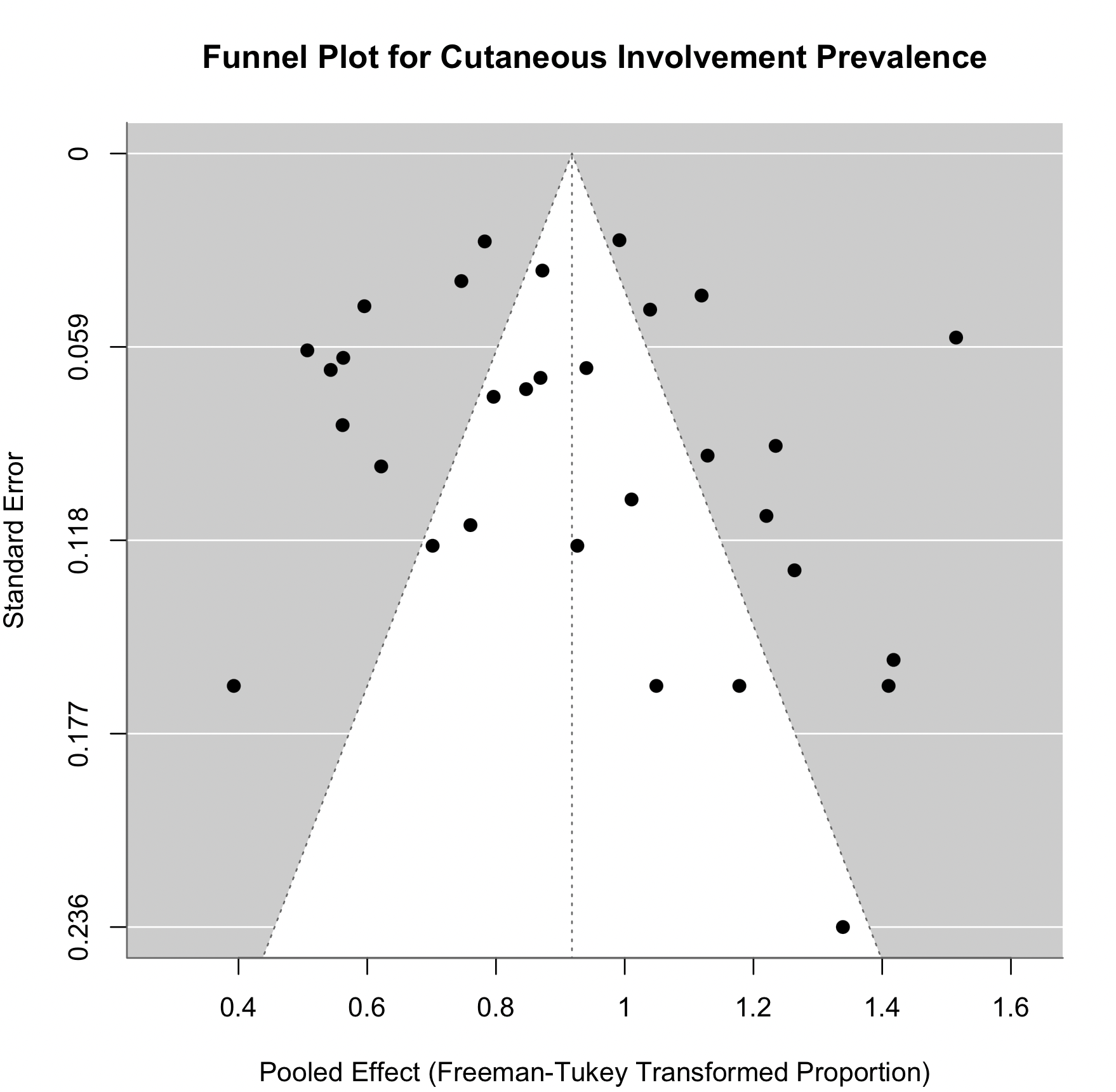


Arthralgia

Model: mixed-effects meta-regression model

Predictor: standard error

Test for Funnel Plot Asymmetry: z = 1.8779, p = 0.0604

Limit Estimate (as sei -> 0): b = 0.6258 (CI: 0.4659, 0.7857)


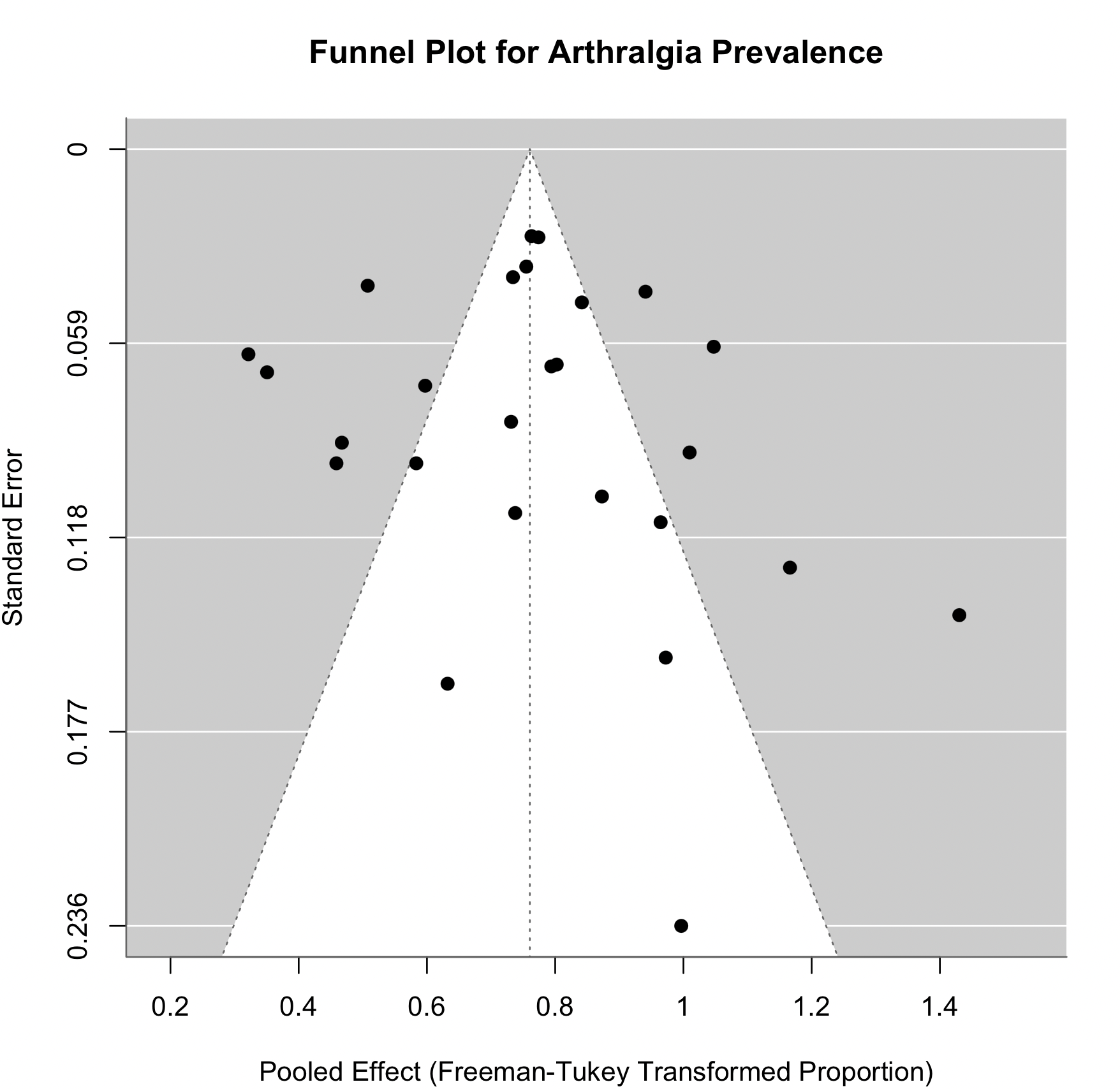


Gastrointestinal Involvement

Model: mixed-effects meta-regression model

Predictor: standard error

Test for Funnel Plot Asymmetry: z = -0.2377, p = 0.8121

Limit Estimate (as sei -> 0): b = 0.6480 (CI: 0.5378, 0.7583)


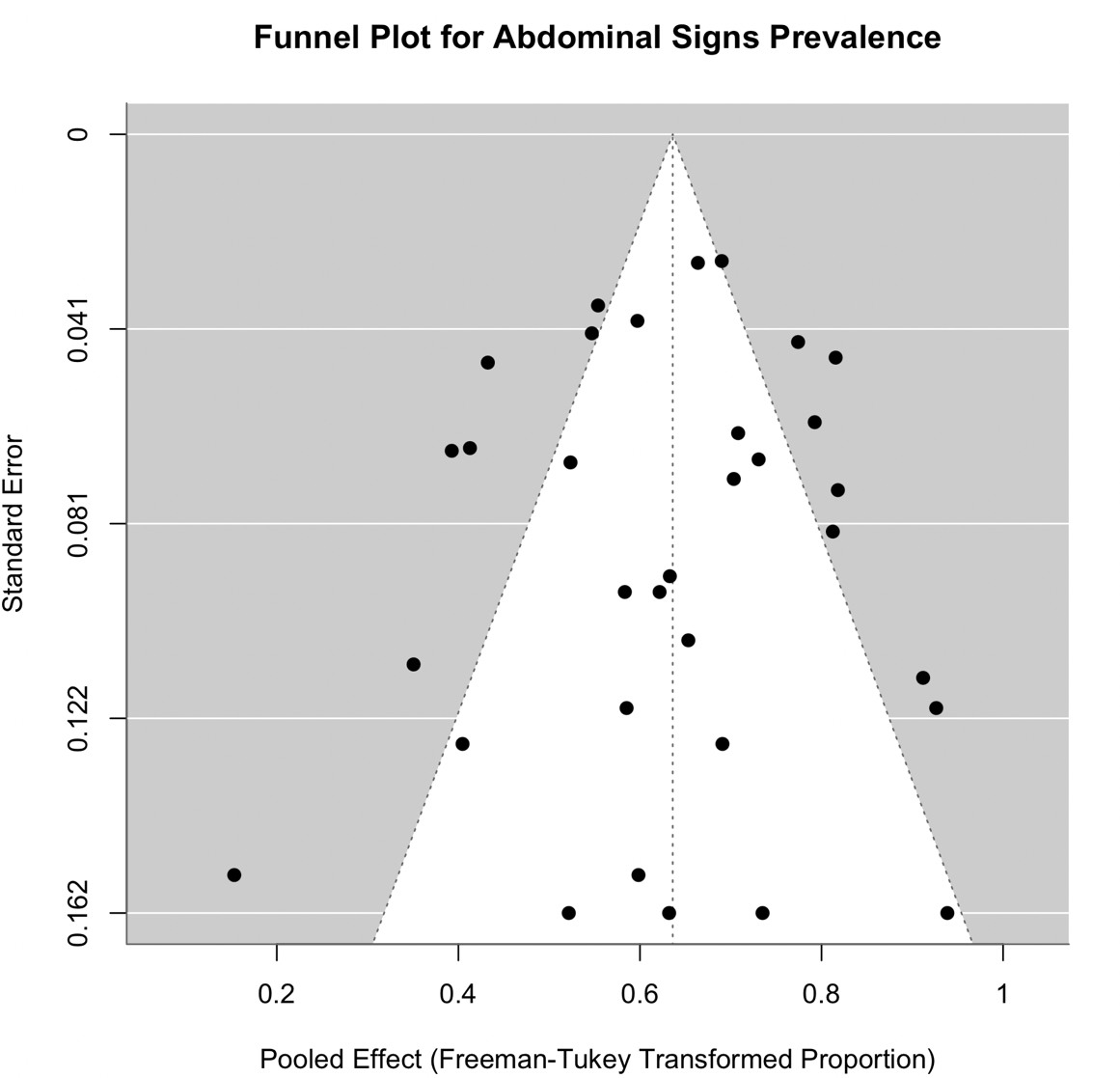


Hypertension

Model: mixed-effects meta-regression model

Predictor: standard error

Test for Funnel Plot Asymmetry: z = 2.4586, p = 0.0139

Limit Estimate (as sei -> 0): b = 0.4051 (CI: 0.2558, 0.5543)


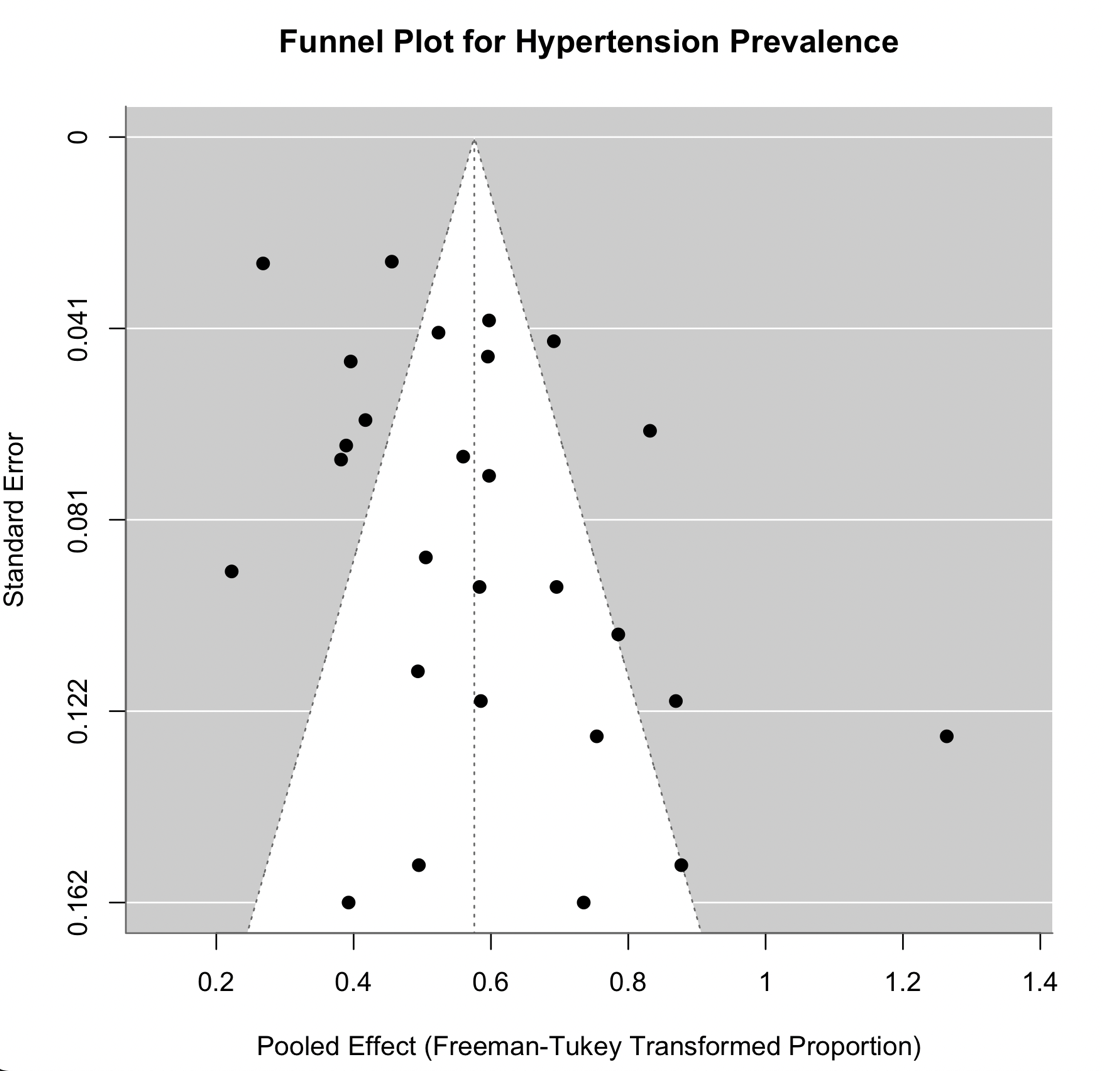


Cardiac Involvement

Model: mixed-effects meta-regression model

Predictor: standard error

Test for Funnel Plot Asymmetry: z = 2.9923, p = 0.0028

Limit Estimate (as sei -> 0): b = 0.1980 (CI: 0.0426, 0.3534)


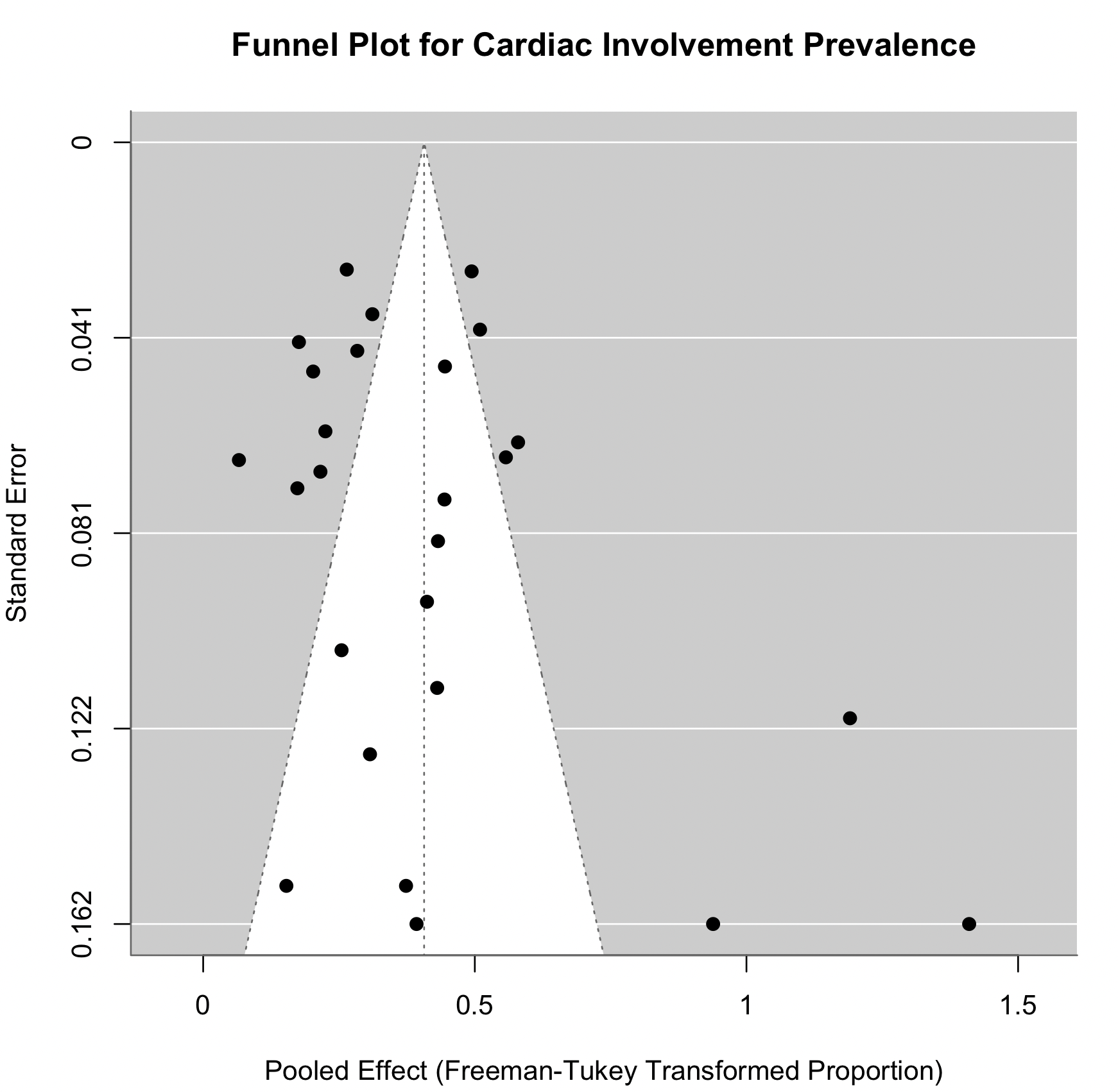


CNS Involvement

Model: mixed-effects meta-regression model

Predictor: standard error

Test for Funnel Plot Asymmetry: z = 0.8699, p = 0.3843

Limit Estimate (as sei -> 0): b = 0.3428 (CI: 0.2342, 0.4514)


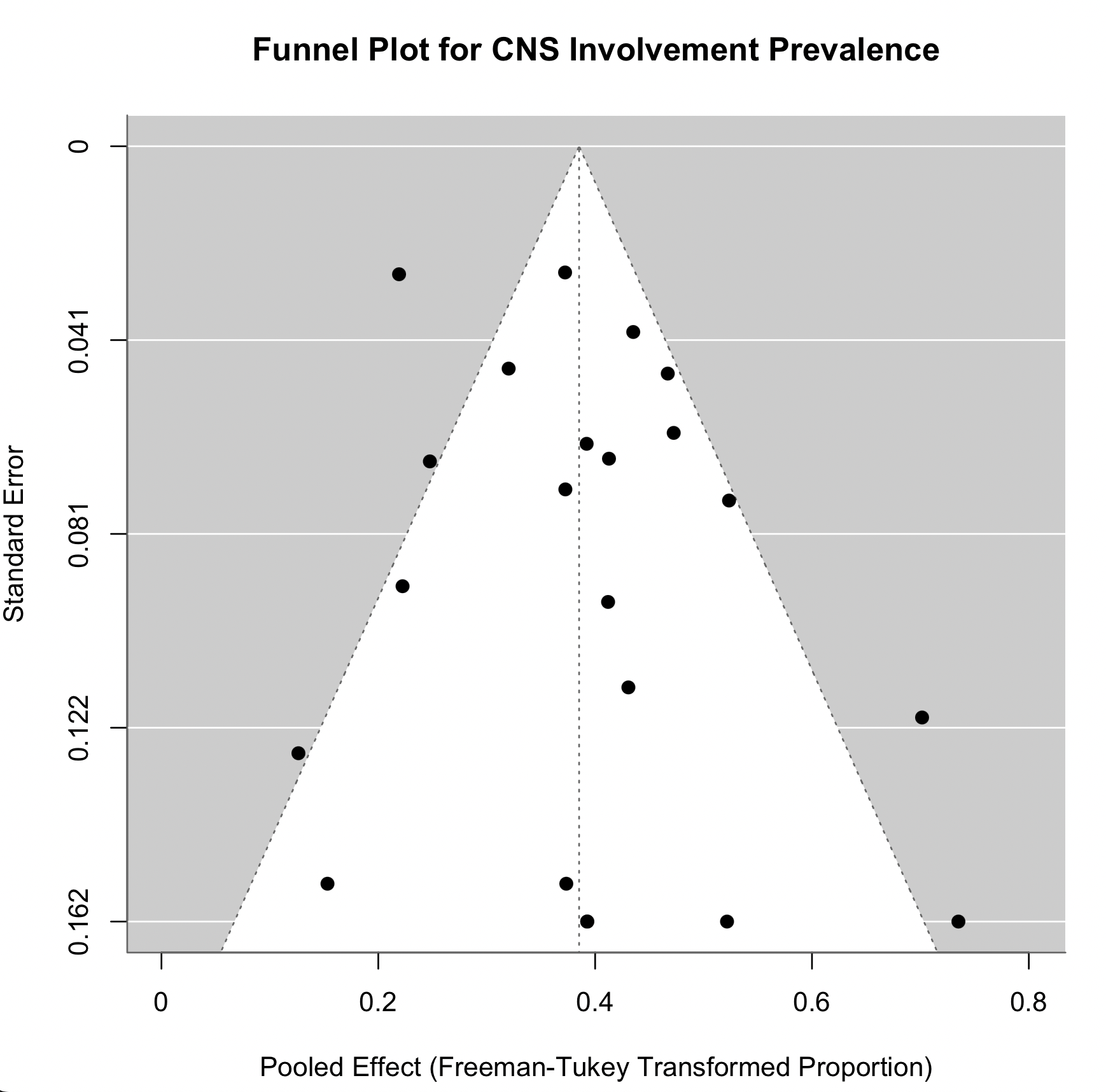


Neuropathy

Model: mixed-effects meta-regression model

Predictor: standard error

Test for Funnel Plot Asymmetry: z = 0.2599, p = 0.7950

Limit Estimate (as sei -> 0): b = 0.5847 (CI: 0.2480, 0.9214)


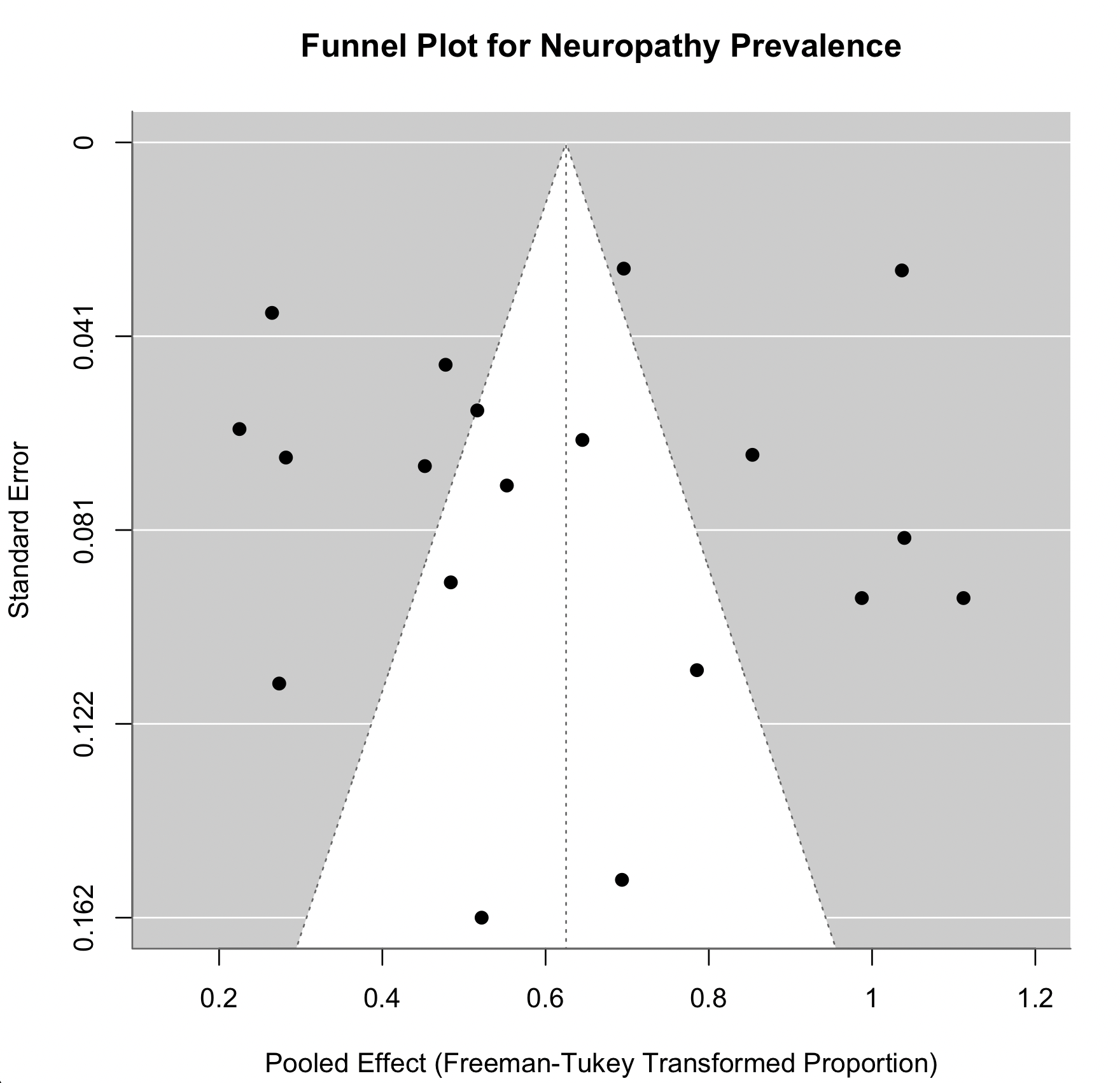


Renal Involvement

Model: mixed-effects meta-regression model

Predictor: standard error

Test for Funnel Plot Asymmetry: z = -0.5489, p = 0.5830

Limit Estimate (as sei -> 0): b = 0.6319 (CI: 0.4581, 0.8057)


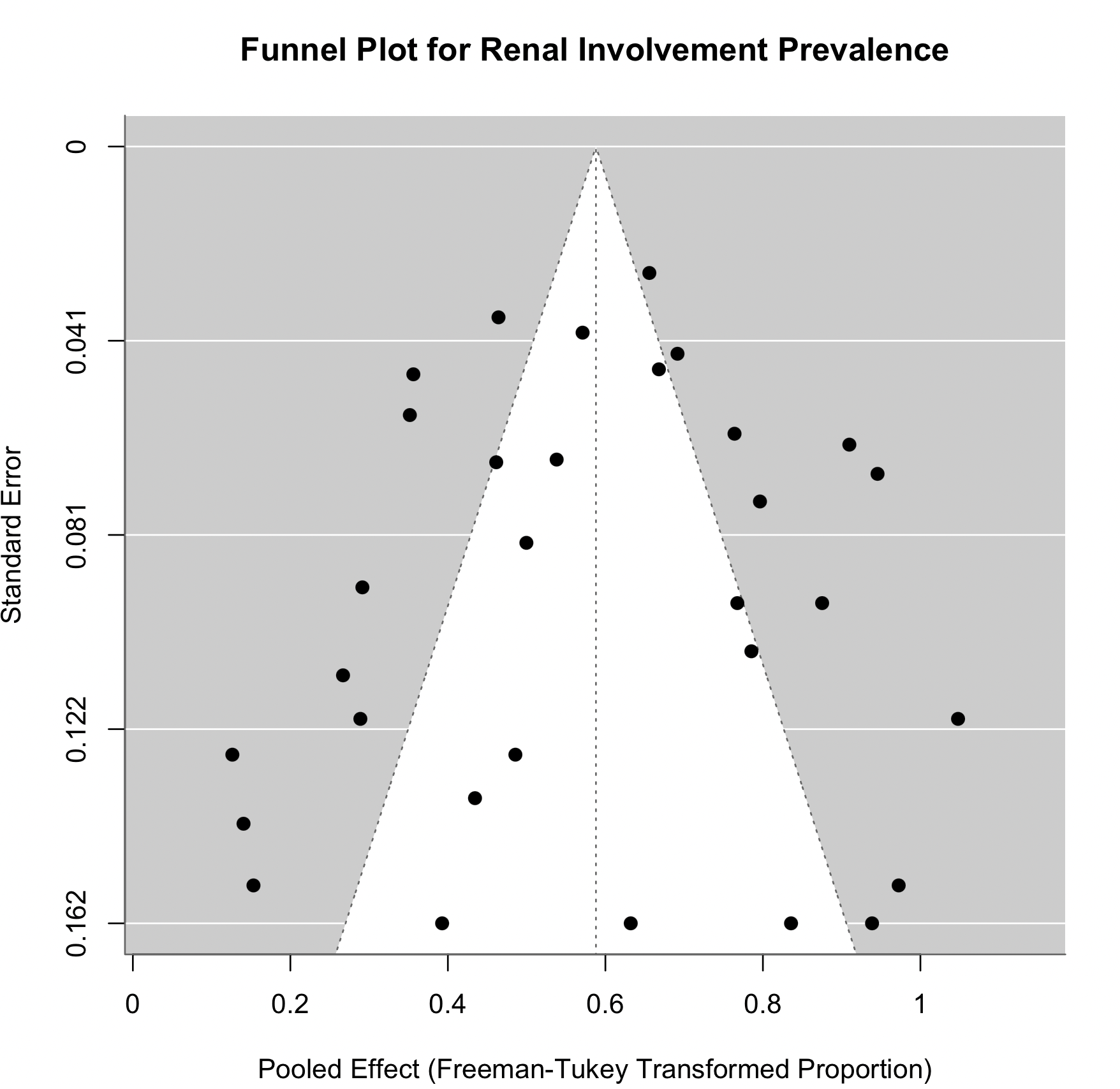


Mortality

Model: mixed-effects meta-regression model

Predictor: standard error

Test for Funnel Plot Asymmetry: z = -0.3172, p = 0.7511

Limit Estimate (as sei -> 0): b = 0.4126 (CI: 0.2604, 0.5648)


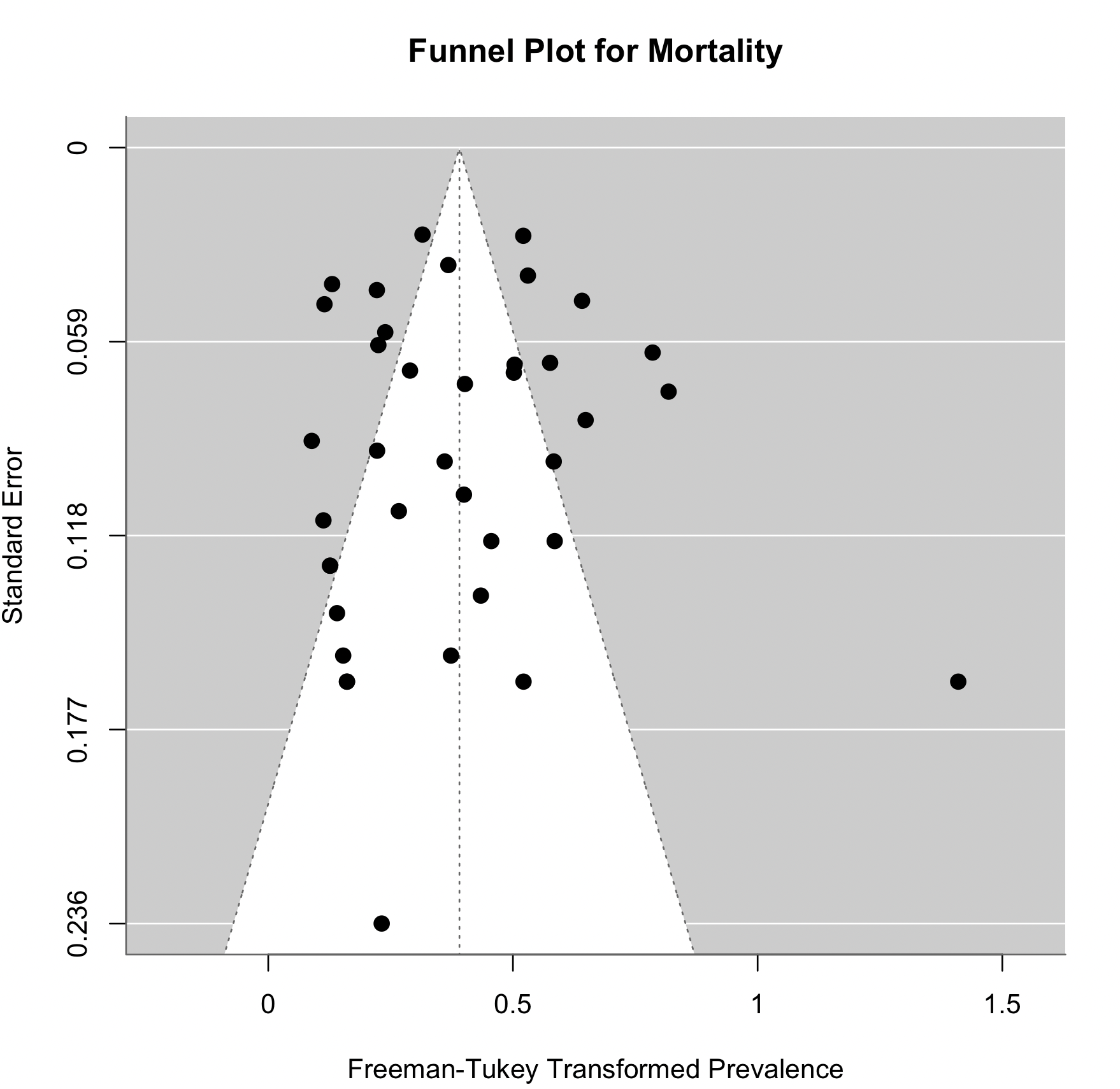


Relapse

Regression Test for Funnel Plot Asymmetry

Model: mixed-effects meta-regression model

Predictor: standard error

Test for Funnel Plot Asymmetry: z = 0.7528, p = 0.4516

Limit Estimate (as sei -> 0): b = 0.4968 (CI: 0.3186, 0.6750)


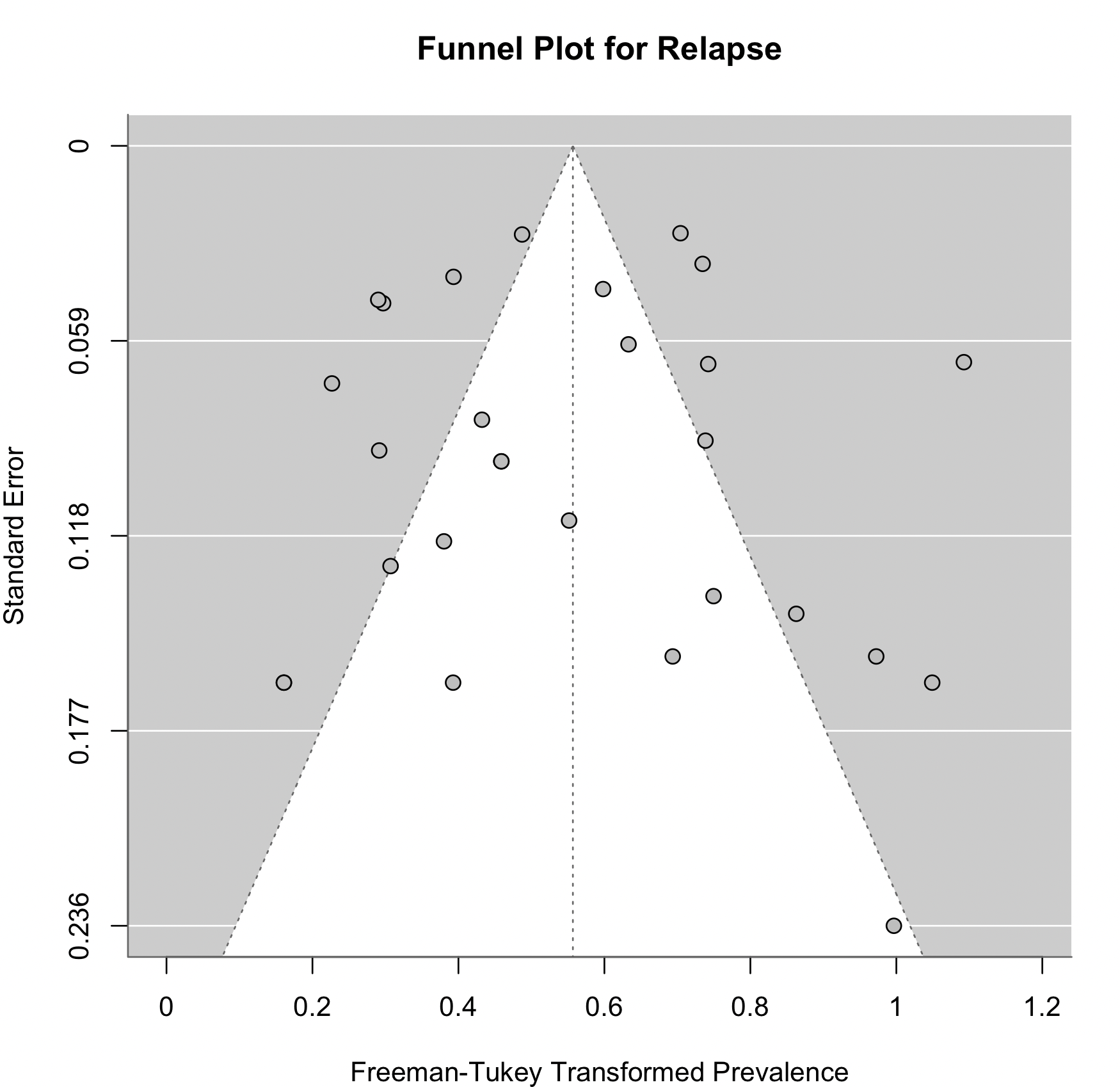


Remission

Model: mixed-effects meta-regression model

Predictor: standard error

Test for Funnel Plot Asymmetry: z = -1.1244, p = 0.2608

Limit Estimate (as sei -> 0): b = 1.0961 (CI: 0.7781, 1.4140)


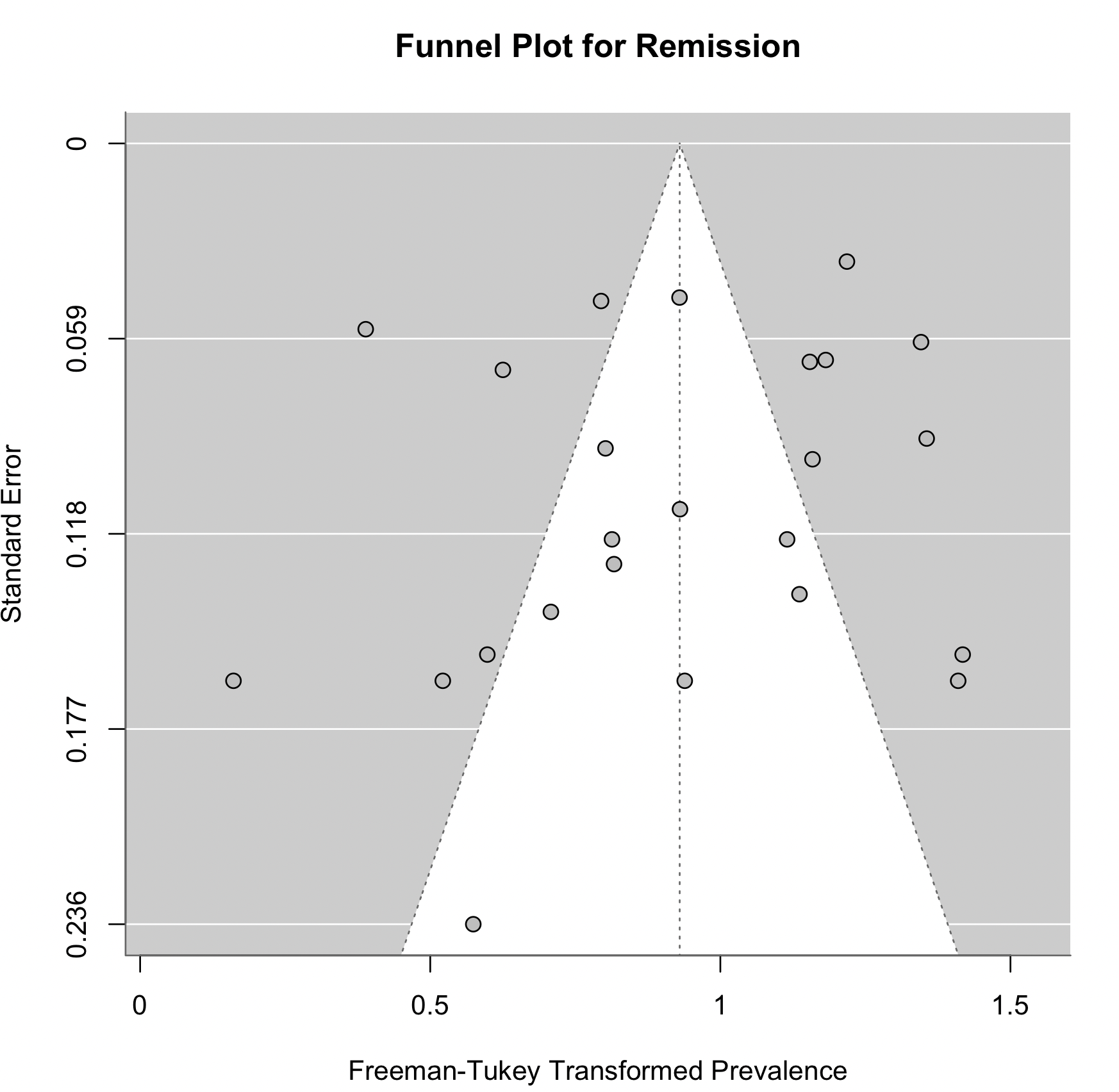

Supplement: Supplementary file 3 — Supplementary Material 3 [file 296_2026_6082_MOESM3_ESM.docx]
