# Supplementary material for: Clinical manifestations, prognostic impact, and relapse in polyarteritis nodosa: a systematic review and meta-analysis
Source: Rheumatol Int. 2026 Feb 19;46(3):51. doi: 10.1007/s00296-026-06082-8 (PMC12920359; doi:10.1007/s00296-026-06082-8)
Supplement: Supplementary file 4 — Supplementary Material 4 [file 296_2026_6082_MOESM4_ESM.docx]

| Parameters | Prevalence in High Risk Studies (%) | Prevalence in Moderate Risk Studies (%) | Prevalence in Low Risk Studies (%) | p-value |
| --- | --- | --- | --- | --- |
| Fever | 68.5 | 60.7 | 58.3 | 0.6970 |
| Weight Loss | 46.9 | 44.2 | 55.9 | 0.5195 |
| Myalgia | 58.0 | 47.1 | 64.0 | 0.2004 |
| Cutaneous Involvement | 69.8 | 69.0 | 47.2 | 0.0886 |
| Arthralgia | 62.3 | 41.4 | 52.3 | 0.2817 |
| Hypertension | 23.1 | 34.7 | 23.4 | 0.2733 |
| Gastrointestinal Involvement | 33.8 | 38.3 | 34.9 | 0.8590 |
| Cardiac Involvement | 51.5 | 11.2 | 10.9 | 0.0896 |
| CNS Involvement | 20.8 | 12.4 | 12.0 | 0.7440 |
| Peripheral Neuropathy | 53.0 | 44.0 | 34.7 | 0.4773 |
| Renal Involvement | 24.5 | 29.2 | 31.9 | 0.8504 |
| Mortality | 16.5 | 11.5 | 13.2 | 0.6863 |
| Relapse | 34.5 | 17.8 | 33.4 | 0.0959 |
| Remission | 50.0 | 73.2 | 78.0 | 0.1059 |

Table 1: Risk of Bias Levels Subgroup Analysis
